# Cymantrenyl-Nucleobases: Synthesis, Anticancer, Antitrypanosomal and Antimicrobial Activity Studies

**DOI:** 10.3390/molecules22122220

**Published:** 2017-12-14

**Authors:** Artur Jabłoński, Karolina Matczak, Aneta Koceva-Chyła, Kamil Durka, Dietmar Steverding, Katarzyna Jakubiec-Krześniak, Jolanta Solecka, Damian Trzybiński, Krzysztof Woźniak, Vanesa Andreu, Gracia Mendoza, Manuel Arruebo, Krzysztof Kochel, Barbara Krawczyk, Dominik Szczukocki, Konrad Kowalski

**Affiliations:** 1 Department of Organic Chemistry, Faculty of Chemistry, University of Łódź, Tamka 12, 91-403 Łódź, Poland; ajablonski88@interia.pl; 2 Department of Medical Biophysics, Faculty of Biology and Environmental Protection, University of Łódź, Pomorska 141/143, 90-236 Łódź, Poland; karolina.matczak@biol.uni.lodz.pl (K.M.); aneta.koceva@biol.uni.lodz.pl (A.K.-C.); kamil.durka@biol.uni.lodz.pl (K.D.); kochel@biol.uni.lodz.pl (Kr.K.); 3 Bob Champion Research & Education Building, Norwich Medical School, University of East Anglia, Norwich Research Park, Norwich NR4 7UQ, UK; D.Steverding@uea.ac.uk; 4 National Institute of Public Health-National Institute of Hygiene, Chocimska 24, 00-791 Warszawa, Poland; kkrzesniak@pzh.gov.pl (K.J.-K.); jsolecka@pzh.gov.pl (J.S.); 5 Biological and Chemical Research Centre, Department of Chemistry, University of Warsaw, Żwirki and Wigury 101, 02-089 Warszawa, Poland; trzybinski@chem.uw.edu.pl (D.T.); kwozniak@chem.uw.edu.pl (K.W.); 6 Department of Chemical Engineering. Aragon Institute of Nanoscience (INA), University of Zaragoza, Campus Río Ebro-Edificio I+D, C/ Poeta Mariano Esquillor S/N, 50018 Zaragoza, Spain; vandreu@unizar.es (V.A.); graciamendoza@gmail.com (G.M.); arruebom@unizar.es (M.A.); 7 Networking Research Center on Bioengineering, Biomaterials and Nanomedicine, CIBER-BBN, 28029 Madrid, Spain; 8 Faculty of Chemistry, Department of Inorganic and Analytical Chemistry, University of Łódź, Tamka 12, 91-403 Łódź, Poland; b.t.krawczyk@gmail.com (B.K.); dominik.szczukocki@gmail.com (D.Sz.)

**Keywords:** cymantrene, nucleobases, bioorganometallics, anticancer activity, antitrypanosomal activity, antibacterial activity

## Abstract

The synthesis of four cymantrene-5-fluorouracil derivatives (**1**–**4**) and two cymantrene-adenine derivatives (**5** and **6**) is reported. All of the compounds were characterized by spectroscopic methods and the crystal structure of two derivatives (**1** and **6**), together with the previously described cymantrene-adenine compound **C** was determined by X-ray crystallography. While the compounds **1** and **6** crystallized in the triclinic P-1 space group, compound **C** crystallized in the monoclinic *P*2_1_/*m* space group. The newly synthesized compounds **1**–**6** were tested together with the two previously described cymantrene derivatives **B** and **C** for their in vitro antiproliferative activity against seven cancer cell lines (MCF-7, MCF-7/DX, MDA-MB-231, SKOV-3, A549, HepG2m and U-87-MG), five bacterial strains *Staphylococcus aureus* (methicillin-sensitive, methicillin-resistant and vancomycin-intermediate strains), *Staphylococcus epidermidis*, and *Escherichia coli*, including clinical isolates of *S. aureus* and *S. epidermidis*, as well as against the protozoan parasite *Trypanosoma brucei*. The most cytotoxic compounds were derivatives **2** and **C** for A549 and SKOV-3 cancer cell lines, respectively, with 50% growth inhibition (IC_50_) values of about 7 µM. The anticancer activity of the cymantrene compounds was determined to be due to their ability to induce oxidative stress and to trigger apoptosis and autophagy in cancer cells. Three derivatives (**1**, **4** and **5**) displayed promising antitrypanosomal activity, with GI_50_ values in the low micromolar range (3–4 µM). The introduction of the 5-fluorouracil moiety in **1** enhanced the trypanocidal activity when compared to the activity previously reported for the corresponding uracil derivative. The antibacterial activity of cymantrene compounds **1** and **C** was within the range of 8–64 µg/mL and seemed to be the result of induced cell shrinking.

## 1. Introduction

Over the last years, organometallic compounds have been studied with respect to various biological applications [1,2,3,4]. The majority of them are related to anticancer therapy [5,6,7,8], while less attention has been devoted to organometallics as antibacterial [9,10] and antiparasite agents [11,12].

Cymantrene (tricarbonyl(η^5^-cyclopentadienyl)manganese; **CymH**) is a half-sandwich organometallic compound that is stable in air and water [13]. The cymantrene molecule can be functionalized at the η^5^-cyclopentadienyl ligand [14,15,16], and at the tricarbonyl ligand position [17,18]. This structural diversity of the cymantrene molecule is the reason for its wide applicability in synthetic chemistry [19,20,21]. Recently, cymantrene derivatives have attracted considerable interest as biologically active agents. For example, they have been reported as markers in immunoassays [22] and as intermediates in the synthesis of organometallic steroid derivatives [23], and the cymantrenyl group has been used for tamoxifen modification [24]. Furthermore, it has been shown that conjugation of cymantrene to cell-penetrating peptides or proteins significantly increase their cytotoxic effect against cancer cells [25,26,27].

Another field, where cymantrene derivatives have been studied, comprises the development of antibacterial and antiparasite agents [28,29,30,31,32,33]. For instance, hetero-tri-organometallic compounds containing the cymantrene moiety showed significant activity against methicillin-resistant *Staphylococcus aureus* (MRSA) strain [30]. Furthermore, cymantrene derivatives of the antibiotic platensimycin were reported [31]. In addition, cymantrene 4-aminoquinoline derivatives have been investigated for activity against the malaria parasite *Plasmodium falciparum*, the sleeping sickness parasite *Trypanosoma brucei*, and the Chagas disease parasite *Trypanosoma cruzi* [32]. Moreover, antitrypanosomal activity was also reported for cymantrene triazole derivatives [33].

The above-mentioned examples support the development of new cymantrene-based compounds with anticancer, antibacterial and antiparasite activity. Of particular importance is the new class of cymantrene-nucleobase conjugates, recently reported by us [14]. Interestingly enough, some of these molecules showed activity against bloodstream forms of *T. brucei* in the micromolar range. In continuation and extension of our long-standing program in the field of bioorganometallic chemistry [34,35,36,37], we describe here the synthesis and biological activity of six new cymantrene-nucleobase (nucleobase = 5-fluorouracil or adenine) conjugates, together with the crystal structures of three of the compounds. The main goal of the work was to evaluate the compounds against a broad range of biological targets. The compounds were investigated for their antiproliferative activity (i) against a panel of human cancer cells; (ii) against the protozoan parasite *T. brucei*; and (iii) Gram-positive and Gram-negative bacteria strains. Moreover, for most of the compounds with anticancer activity, additional biological assays were conducted in order to elucidate their molecular mechanisms of action.

## 2. Results and Discussion

### 2.1. Synthesis of Compounds ***1***–***7***

**Scheme 1:**
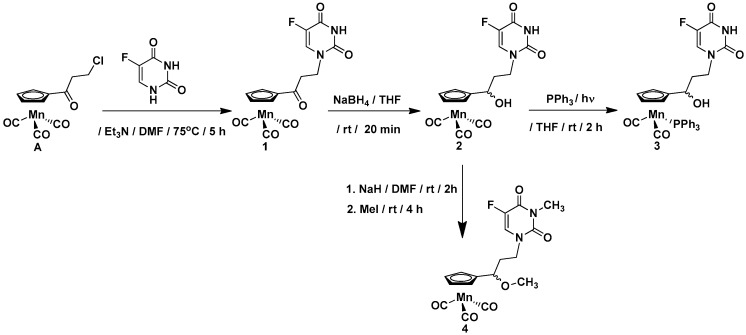
Synthesis of cymantrene-5-fluorouracil derivatives **1**–**4**.

**Scheme 2:**
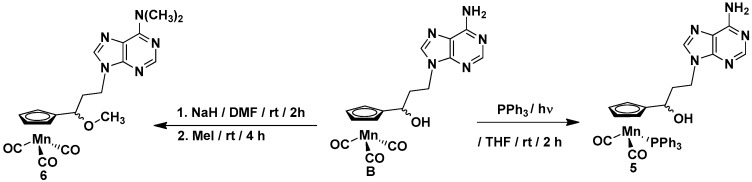
Synthesis of cymantrene-adenine derivatives **5** and **6**.

The compounds synthesized during this work can be divided into two groups. The first group includes the cymantrene-5-fluorouracil derivatives **1**–**4**, while the second group comprises the cymantrene-adenine derivatives **5** and **6**. This selection was justified by previous results, showing that some ferrocene-5-fluorouracil and cymantrene-adenine conjugates displayed activity against *S. aureus* (MRSA) and *T. brucei* pathogens, respectively [14,36]. The synthetic approach for the preparation of **1**–**4** is shown in Scheme 1, whereas the synthesis of **5** and **6** is depicted in Scheme 2.

In general, the synthesis exploited methodologies developed recently in our laboratory for ferrocene, ruthenocene, and [2.2] paracyclophane nucleobase derivatives [36,37]. In a first step, 3-chloropropionylocymantrene **A** [14] reacted with 5-fluorouracil to afford ketone **1** in 67% yield. In a second step, the carbonyl group in **1** was reduced with sodium tetrahydridoborate to afford alcohol **2** in 85% yield.

To obtain the products **3** and **5**, the photochemical substitution reaction of the carbonyl ligand in alcohol **2** (Scheme 1) or alcohol **B** (Scheme 2) by the triphenylphosphine was utilized. Accordingly, compounds **3** and **5** were obtained in 47% and 43% yields, respectively. The subsequent treatment of the alcohols **2** and **B** with sodium hydride and methyl iodide allowed for obtaining the methylated compounds **4** and **6** in 40% and 79% yields, respectively. For assessing the biological activity of the newly obtained compounds, the propionylocymantrene **7** was also synthesized through Friedel-Crafts reaction and was fully characterized (Figure S4 and Scheme S1 in the SI).

The compounds **1**, **2**, and **7** are yellow solids, the complexes **3** and **5** are green solids, and compound **6** is a colorless solid, while compound **4** is a yellow oil. The entire series of compounds is air-stable and can be stored in the fridge for months without signs of decomposition. The products were characterized by ^1^H-NMR, ^13^C-NMR, IR, mass spectrometry (MS), and elemental analysis.

### 2.2. X-ray Crystal Structures of ***1***, ***6*** and ***C***

**Figure 1:**
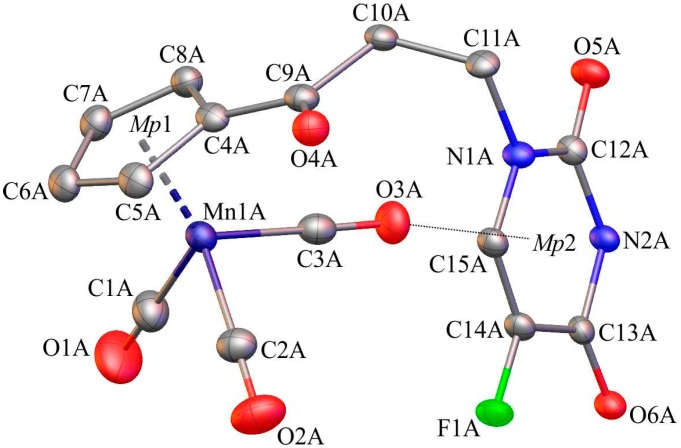
The molecular diagram of **1** with atomic displacement ellipsoids at the 50% probability level; Mp1 corresponds to the midpoint of the cyclopentadienyl ring. Hydrogen atoms have been omitted for clarity. Only molecule **1A** is shown. Selected bond lengths [Å] and angles [°]: Mn1(A)-C1(A), 1.822(4); Mn1(A)-C2(A), 1.803(4); Mn1(A)-C3(A), 1.796(3); Mn1(A)-C4(A), 2.131(3); O1(A)-C1(A), 1.126(5); O2(A)-C2(A), 1.147(5); O3(A)-C3(A), 1.150(4); C4(A)-C9(A), 1.472(4); C9(A)-O4(A), 1.220(4); C10(A)-C11(A), 1.532(4); N1(A)-C12(A), 1.369(3); C13(A)-O6(A), 1.222(4); C13(A)-N2(A), 1.385(3); C14(A)-F1(A), 1.352(3); C5(A)-C4(A)-C9(A)-O4(A); −7.4(5); and, N1(A)-C12(A)-N2(A)-C13(A), 6.9(4).

**Figure 2:**
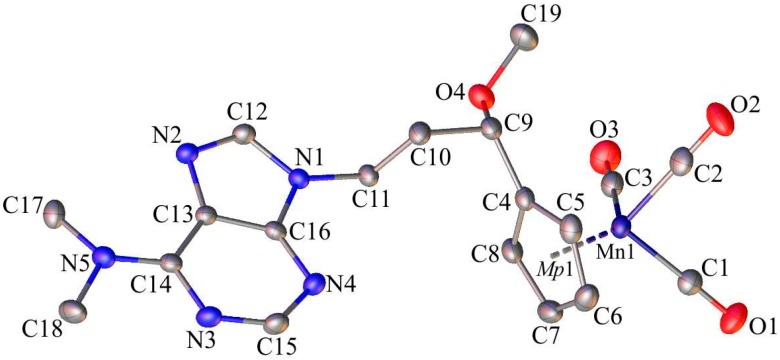
The molecular diagram of **6** with atomic displacement ellipsoids at the 50% probability level; Mp1 corresponds to mid-point of the cyclopentadienyl ring. Hydrogen atoms have been omitted for clarity. Selected bond lengths [Å] and angles [°]: Mn1-C1, 1.795(2); Mn1-C2, 1.789(2); Mn1-C3, 1.797(2); O1-C1, 1.151(2); O2-C2, 1.152(3); O3-C3, 1.150(3); C4-C9, 1.503(2); C9-O4, 1.412(2); C19-O4, 1.423(2); C10-C11, 1.522(2); N1-C12, 1.368(2); N2-C13, 1.396(2); N5-C14, 1.353(2); C17-N5, 1.460(2); C5-C6-C7-C8; 0.3(2); N1-C12-N2-C13; −0.4(2); N3-C15-N4-C16; and, 1.1(3).

**Figure 3:**
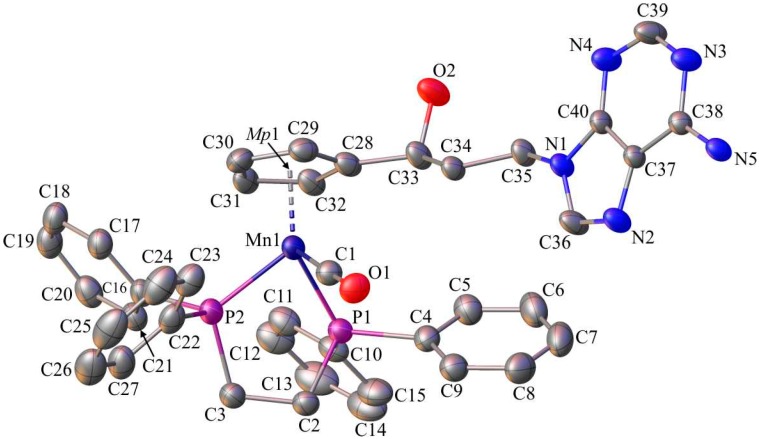
The molecular diagram of **C** with atomic displacement ellipsoids at the 50% probability level; Mp1 corresponds to mid-point of the cyclopentadienyl ring. Hydrogen atoms and solvent molecules have been omitted for clarity. Selected bond lengths [Å] and angles [°]: Mn1-C1, 1.760(3); Mn1-P1, 2.199(1); Mn1-P2, 2.193(1); O1-C1, 1.169(4); P2-C3, 1.862(3); P1-C2, 1.854(3); C2-C3, 1.540(4); C28-C33, 1.502(4); C33-O2, 1.417(4); C34-C35, 1.516(4); N1-C36, 1.356(4); N2-C37, 1.379(4); N5-C38, 1.327(4); P1-C2-C3-P2, −40.0(3); C29-C30-C31-C32, 0.3(4); N1-C36-N2-C37, 0.1(4); and, N3-C39-N4-C40, 0.0(7).

Single-crystals of **1**, **6**, and **C** suitable for X-ray diffraction analysis were crystallized by slow diffusion of pentane into chloroform solutions of **1**, **6**, and **C**. The molecular diagrams of the molecules **1**, **6**, and **C**, with the atom labelling schemes are shown in Figure 1, Figure 2 and Figure 3 together with selected numerical values for bond lengths and valence angles. Crystal and structure refinement details are given in Table S1. The bond lengths and valence angles are summarized in Tables S2–S7. Compounds **1** and **6** crystallized in the triclinic *P*-1 space group, while compound **C** crystallized in the monoclinic *P*2_1_/*n* space group. In the crystal lattice of **1**, two independent molecules (**1A** and **1B**) were observed. The compound **C** crystallizes as a solvate with two chloroform molecules in the asymmetric part of the unit cell. The molecular drawing of the solvate is provided in Figure S10. The X-ray crystal structure analysis of compounds **1**, **6**, and **C** confirmed that the cymantrenyl moiety had a three-legged piano-stool structure. The distance between the Mn-atom and the midpoint (Mp1) of the cyclopentadienyl ring was 1.771(2) Å for **1A** and **6**, 1.770(2) Å for **1B** and 1.767(2) Å for **C**. These values are close to that of 1.764(3) Å reported previously for compound **B** [14].

The differences in the corresponding geometrical parameters of **1A** and **1B** were small and they were related to the slightly altered orientation of the 5-fluorouracil group relative to the cymantrenyl moiety. In both of the molecules, the plane of the 5-fluorouracil moiety was almost perpendicular relative to the plane of the cyclopentadienyl ring. A dihedral angle between the above-mentioned fragments in **1A** and **1B** was 97.40(2)° and 87.61(2)°, respectively. In both molecules, the 5-fluorouracil moiety was directed toward the oxygen atom of one of the carbonyl ligands. The distance between the calculated 5-fluorouracil ring midpoint (Mp2) and the O3A atom in **1A** was 3.040(3)Å, while the corresponding distance in the conformer **1B** was 3.138(3)Å, respectively. In the case of compound **C**, the X-ray diffraction analysis confirmed that the 1,2-bis(diphenylphosphino)ethane ligand (dppe) binds to the Mn-atom in a chelate fashion. The plane of the adenine moiety was almost perpendicular to the plane of the cyclopentadienyl group. The angle between these two planes was 90.73(11)°. In the solid state, individual molecules of compound **C** were stabilized by intramolecular C35–H35···O2 (2.740(4)Å) hydrogen bond. Furthermore, there also were C31–H31···π-electron interactions between a proton on the cyclopentadienyl ring and one of the phenyl rings in the dppe ligand.

### 2.3. Determination of log P_o/w_

Lipophilicity is an important parameter in the context of biological activity studies and can be expressed by the octanol/water partition coefficient (log *P_o/w_*). In this study, a chromatographic method and the Soczewiński–Wachtmeister relationship were used in order to determine the lipophilicity of the compounds [38,39,40,41] (Table 1). All of the tested compounds showed a good linear correlation with high coefficients of determination (*R*^2^) (Table S8). The partition coefficients of the compounds were within the range of 2.6–5.7. Thus, all of them showed some degree of lipophilicity. The derivative **C** with the dppe ligand was the most lipophilic compounds (log *P_o/w_* = 5.7), while compounds **1** and **B** were the least lipophilic agents (both log *P_o/w_* = 2.6). Since the difference in the partition coefficient of these compounds is greater than three logs, quite different cellular accumulation of these molecules can be expected. In turn, the compound **Cym H** and derivatives **4** and **6** should have similar cellular accumulation patterns as their log *P_o/w_* had the same value (3.5). However, it should be pointed out that lipophilicity is not the sole factor that determines the uptake and distribution of molecules in cells.

## 3. Biological Studies

**Figure 4:**
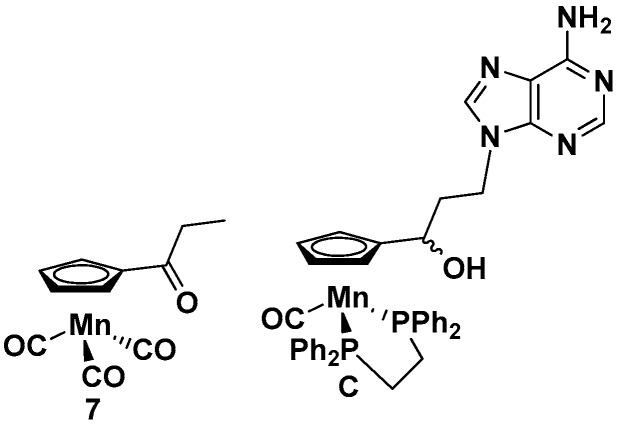
Structures of **7** and **C**.

The newly synthesized compounds **1**–**6** were tested for their anticancer, antitrypanosomal, and antibacterial activities. For comparison, the nucleobase-free compound **7** and the previously studied cymantrene-adenine compound **C** (Figure 4) [14] were also investigated.

### 3.1. Evaluation of Anticancer Activity

Anticancer activity of cymantrenes was determined using a panel of human cancer cell lines that were derived from solid tumors. These included cell lines of estrogen-responsive, doxorubicin resistant, and triple negative breast adenocarcinomas (MCF-7, MCF-7/DX, and MDA-MB-231), ovarian adenocarcinoma (SKOV-3), non-small cell lung carcinoma (A549), hepatocellular carcinoma (HepG2), and glioblastoma (U-87 MG). The inhibitory effect of cymantrene derivatives on cell proliferation and growth was determined based on survival curves and was expressed as IC_50_ values (concentration required to achieve a 50% decrease in cell viability when compared to the viability of untreated control cells) calculated using GraphPad Prism 4.02 software for Windows (GraphPad Software, San Diego, CA, USA). Cell viability was determined by the MTT microplate spectrophotometric cytotoxicity test.

Most compounds showed a dose-dependent effect on the growth of the cancer cell lines assayed (Figure S11A,B), with most IC_50_ value in the mid micromolar range (Table 1). The most active compounds were the cymantrene-5-fluorouracil derivative **2** for A549 cells and the cymantrene-adenine derivative **C** for SKOV-3 cells with IC_50_ concentration of around 7 µM. Interestingly, the replacement of a CO group in **2** with a triphenylphosphine group in **3** increased the cytotoxic activity against most cell lines (apart form A549) (Table 1). However, the double methylated derivative of **2**, was inactive up to 150 µM (the highest concentration employed) against all of the cell lines tested (Table 1). Another observation is that the keto derivative of compound **2**, compound **1**, displayed better cytotoxic activity for all but one of the cell lines (Table 1). A similar effect, which reduced activity upon reduction of a keto function to a hydroxyl function, was previously observed for ferrocenyl nucleobase derivatives [35]. Compound **B**, which is the adenine derivative of **2**, did not display any activity against any cell line (Table 1). However, the methylated derivative of **B**, compound **6**, displayed increased cytotoxic activity against most of the cancer cell lines (except MDA-MB-231) (Table 1). The exchange of one CO group in **B** with a triphenylphosphine group to yield compound **5** improved the cytototoxicity further (Table 1). In general, it can be noted that the phosphine derivatives **3**, **5**, and **C** were the most active compounds towards all cancer cell lines that were studied. These compounds were also the most lipophilic derivatives based on their log *P_o/w_* values, indicating the importance of molecule lipophilicity for the biological activity of cymantrene-nucleobase conjugates. Interesting is also the finding that the unsubstituted cymantrene (**CymH**) and the propionyl-substituted cymantrene (**7**), which both are precursor molecules for the cymantrene nucleobase conjugates, were moderately cytotoxic (Table 1). This shows that the source molecules inherently have cytotoxic properties. Other intriguing observations were that the doxorubicin-resistant MCF-7/DX cell line was more sensitive to compound **3** than its DOX-sensitive counterpart cell line MCF-7. At the same time, an anticancer drug doxorubicin (DOX), which is routinely employed in clinical oncology and was used in our study as a reference, was almost 100-fold less effective against MCF-7/DX cells than to its MCF-7 cells. Moreover, the triple negative MDA-MB-231 cell line was also more sensitive to the most of the evaluated cymantrene derivatives than the hormone-dependent MCF-7 cell line, which is the advantage of the tested compounds. In this respect, one has to remember that new strategies in the treatment of triple-negative breast cancer, which is one of the most refractory cancer to conventional chemotherapy, are highly required [42]. Our results show that under the same conditions, the reference drug DOX was about twofold less effective against MDA-MB-231 cells than against MCF-7 cells. Among investigated cancer cell lines, non-small lung adenocarcinoma cells A549 were the most sensitive to the majority of the investigated compounds.

### 3.2. Determination of Cytotoxic Mechanism of Action

To get a deeper insight into the molecular mechanisms that are underlying the anticancer activity of the studied cymantrenes, we investigated their ability to generate oxidative stress (OS) and evaluated the type of cell death that was triggered in treated cancer cells. Experiments were performed with the ovarian adenocarcinoma SKOV-3 cells because this cell line was the most sensitive to the overall most active compound, the adenine conjugate **C**.

#### 3.2.1. Induction of Apoptosis and Autophagy

**Figure 5:**
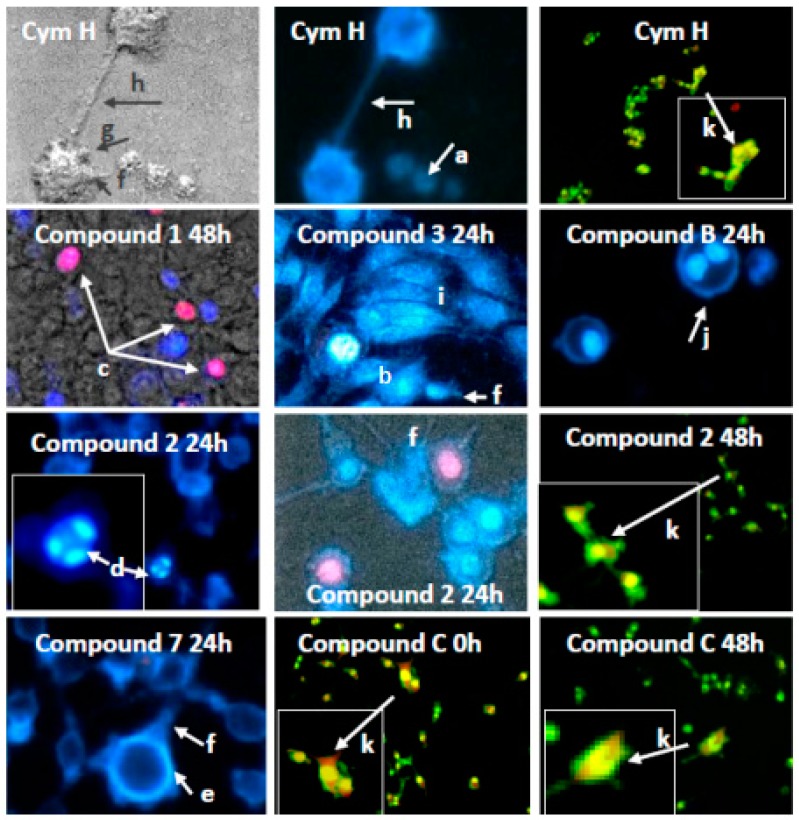
Example enlarged photomicrographs illustrating induction of apoptosis, autophagy and mitotic catastrophe in human ovarian adenocarcinoma cells SKOV-3 by cymantrene derivatives **1**, **2**, **3**, **7**, **B**, and **C** and their core precursor **CymH**. The cells were stained with acridine orange (AO) and double-stained with fluorescence dyes Hoechst 33258 (Ho33258) and propidium iodide (PI) and then analyzed with an inverted fluorescence microscope (Olympus IX70, Tokyo, Japan) at 400× magnification, except cells stained with AO, which were photographed under 150× magnification. The images marked with arrows show the most typical morphological changes associated with different type of cell death induced by cymantrene compounds during 24 h-treatment and following culture of SKOV-3 cells in drug-free medium for 24 or 48 h (0 h—cells examined immediately after the treatment): (**a**) pale-blue live cells; (**b**) intense bright-blue early apoptotic shrunk cells with pycnotic nucleus; (**c**) violet late apoptotic cells; (**d**) cells with highly condensed and fragmented chromatin (karyorrhexis)**;** (**e**) marginalization of chromatin; (**f**) cells with plasma membrane protrusions (“blebs”); (**g**) apoptotic bodies; (**h**) cytoplasmic bridges between the cells. Presence of giant cells (**i**), polyploid cells with two nuclei (**j**) and cells with AVOs (**k**) suggest concomitant mitotic catastrophe and autophagy, respectively.

To determine the mode of cell death that was triggered by cymantrene derivatives, appropriately stained SKOV-3 cells were evaluated for morphological changes using fluorescence microscopy. For evaluation of apoptosis and autophagy, cells were stained with Hoechst 33258/propidium iodide to distinguish between live, apoptotic and necrotic cells and reveal condensed chromatin and apoptotic bodies (a hallmark of apoptosis), and with acridine orange to detect the development of acidic vesicular organelles (AVOs—a hallmark of autophagy). All cymantrene derivatives mainly induced apoptosis and autophagy (representative examples of these processes are shown in Figure 5, and a full panel of pictures is shown in Figure S12A–C).

**Figure 6:**
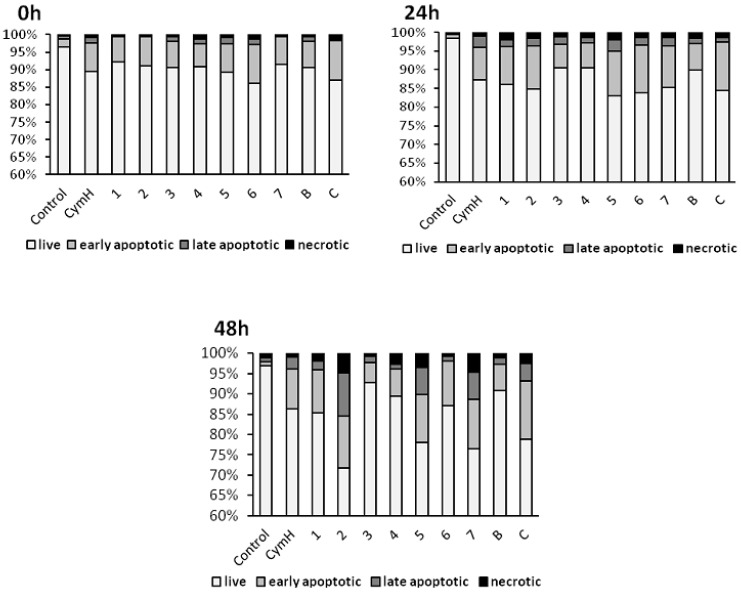
Induction of apoptosis in human ovarian adenocarcinoma cells SKOV-3 by cymantrene derivatives **1**–**7**, **B**, **C**, and their core precursor **CymH**. Changes in the fraction of live, early apoptotic, late apoptotic and necrotic fraction of SKOV-3 cells after 24 h treatment with IC_50_ concentrations of cymantrenes and following post-treatment incubation in drug-free medium for 24 and 48 h.

Numerous morphological changes typical for apoptosis can be identified in cells that are treated with cymantrene compounds: shrunk cells with picnotic nuclei and highly condensed and fragmented chromatin (karyorrhexis), cells with marginalization of chromatin, cells with plasma membrane protrusions (“blebs”), cells with apoptotic bodies, and cells with cytoplasmic bridges between them (Figure 5 and Figure S12A–C). Moreover, cells treated with some of the compounds, e.g., compound **3**, showed features of mitotic cell death, such as the presence of giant and polyploid cells. These observations suggest that induction of apoptosis and possibly mitotic catastrophe could be important mechanisms of the cytotoxic action of cymantrene compounds. However, the different cymantrene derivatives differed in their effectiveness to induce apoptosis in SKOV-3 cells. Time course experiments revealed that, when compared to untreated control cells, cymantrene compounds **3**, **4**, and **B** caused the smallest change (only a three-fold increase of apoptotic cells), while the remaining derivatives produced substantially larger alterations (6-fold (**1**, **6**), 9-fold (**5**, **7**, **C**), and 11-fold (**2**) increase of apoptotic cells) (Figure 6).

While the increase of the apoptotic cell fraction after incubation with compounds **5** and **C** was predominantly related to early apoptotic cells, treatment with compounds **2** and **7** resulted in a rise in the late-apoptotic cell fraction (Figure 6). Bearing in mind that compounds **4** and **B** did not display any cytotoxic activity (IC_50_ > 150 µM), their poor capacity to induce aspoptosis is therefore not surprising. However, that compound **3**, which was the second most cytotoxic cymantrene against SKOV-3 cells, showed only weak apoptotic activity comparable to that of the non-cytotoxic compounds **4** and **B** was unexpected. Nevertheless, it should be pointed out that compound **3** induced extensive mitotic cell death (Figure 5 and Figure S12A–C) that might be responsible for the observed cytotoxic activity of this derivative. At the end of the time course experiment (48 h post-treatment time point), an increase in the fractions of late apoptotic and necrotic cells was noted for all of the compounds (Figure 6). This suggests that some of the damaged apoptotic cells, in particular those treated with compounds **2**, **5**, and **7**, underwent secondary necrosis, contributing to the increase in the necrotic cell fraction. However, necrosis accounted only to a lesser extent to the cytotoxic action of the compounds, which is desirable, as this would reduce the risk of triggering inflammatory processes in neighboring tissue [43].

**Figure 7:**
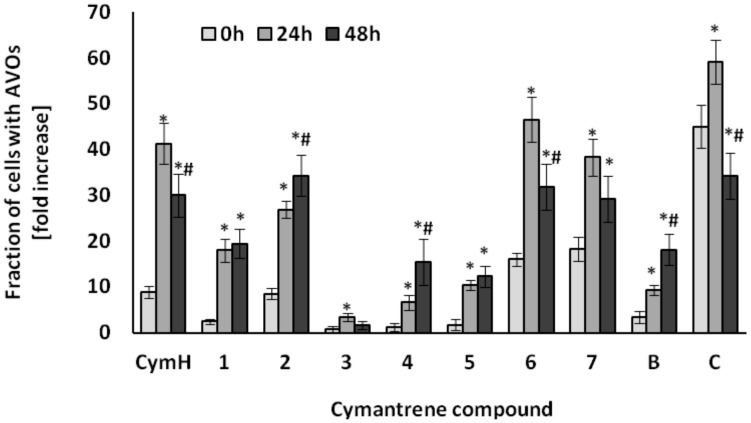
Induction of AVOs in SKOV-3 cells by cymantrene derivatives **1**–**7**, **B**, **C** and **CymH**. The cells were incubated with IC_50_ concentrations of cymantrenes for 24 h under the same conditions as those applied in the cytotoxicity test and then examined immediately after the treatment (0 h time point) or cultured in drug-free medium for 24 or 48 h (24 h and 48 h time points, respectively). The time dependence of changes was statistically evaluated as described in Experimental Section (Section 4.5.2. Statistical Analysis). The fractions of AVOs containing cells at 24 h and 48 h time points were compared with corresponding fractions of AVOs containing cells at 0 h time point (* *p* < 0.05) and the fractions of AVOs containing cells at 48 h time point were compared with corresponding fractions of AVOs containing cells at 24 h time point (# *p* < 0.05). All of the changes in cells treated with cymantrenes were statistically significant in relation to untreated controls (for clarity not shown on the graph).

In addition to apoptosis and mitotic cell death, the process of autophagy was also observed in SKOV-3 cell upon treatment with cymantrene compounds (Figure 5 and Figure S12A–C). Time course experiments showed a significant increase in cells containing AVOs (Figure 7).

The greatest autophagic changes were recorded with the compounds **CymH**, **2**, **6**, **7**, and **C** (Figure 7). Less induction of autophagy was observed with compounds **1**, **4**, **5**, and **B**, while very little autophagic activity was triggered by compound **3** (Figure 7). As the unsubstituted cymantrene molecule was itself a strong inducer of autophagy in SKOV-3 cells, it can be suggested that the cymantrene core of the nucleobase conjugates is probably the autophagy-inducing part of the derivatives. These results indicate that autophagy could be another cytotoxic mechanism of action of cymantrene nucleobase conjugates. The autophagic pathway of cell death has been recently considered as a new important target mechanism that could be exploited to mitigate or to overcome the resistance of tumor cells to chemotherapy-induced apoptosis [44]. Many studies have shown that there is an interplay between apoptosis and autophagy, indicating that the induction of apoptosis is often connected with an activation of autophagy [45]. Depending on the conditions, autophagy may either result in tumor cell death or promote tumor cell survival. For example, following physicochemical cell damages by radiation therapy or chemotherapy, an increased level of autophagy improves the ability of tumor cells to repair the damage [46]. However, excessive autophagy in tumor cells promotes autophagic cell death, resulting in an effective treatment of cancer [47]. Thus, induction of autophagic cell death has been considered in recent times as an important cancer therapeutic strategy. In this regard, the ability of cymantrene compounds to induce both apoptotic and non-apoptotic (pro-death autophagy) death pathways in cancer cells should be seen as a great advantage of this class of organometallics.

#### 3.2.2. Induction of Oxidative Stress

**Figure 8:**
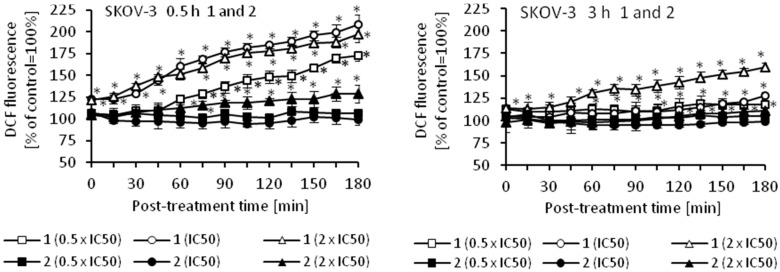

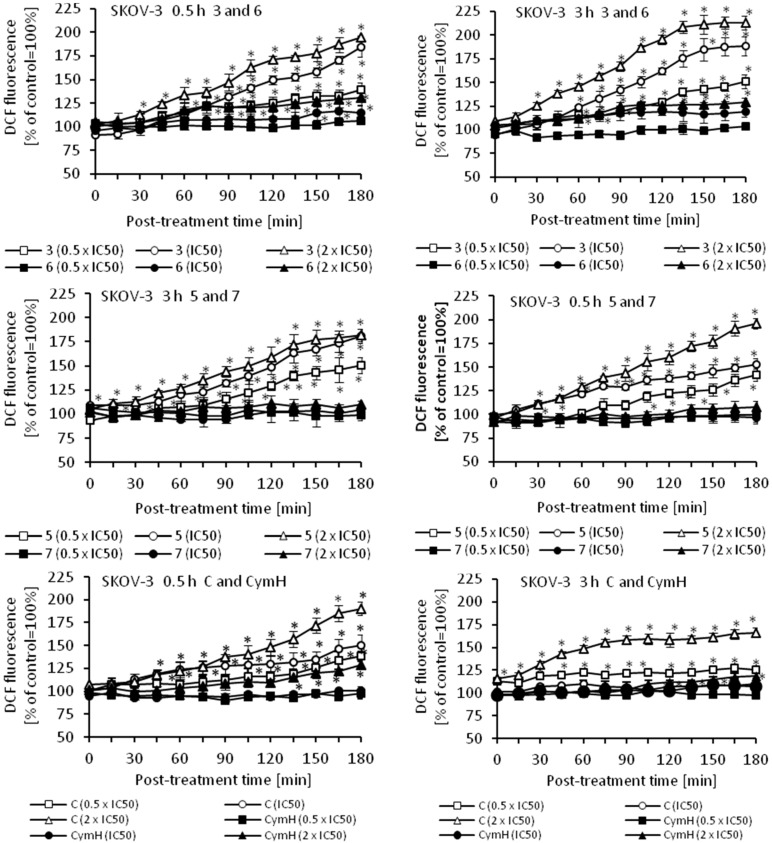
Changes in the relative level of ROS in SKOV-3 cells treated for 0.5 or 3 h with different concentrations (0.5 × IC_50_, IC_50_, and 2 × IC_50_) of cymantrenes **1**, **2**, **3**, **5**, **6**, **7**, **C**, and **CymH** when compared with the ROS amount in untreated controls (100%). The kinetics of ROS formation was chased over 0–180 min time period after the treatment. The results are the means ± SD of 3 independent experiments, each performed in at least six repeats; * *p* < 0.05 as compared to untreated SKOV-3 cells (control).

Oxidative stress is a part of the cytotoxic activity of many anticancer agents and the production of reactive oxygen species (ROS) is considered as one of the main molecular mechanisms accompanying chemotherapy, radiotherapy, and photodynamic therapy [48,49,50,51]. Since ROS can act as both oxidants and antioxidants, depending on the conditions, pro- and anti-oxidant therapies have been proposed for the treatment of cancer. So far, both ROS scavenging and ROS boosting anticancer therapies have shown promising results in vitro and in vivo [48]. Using an appropriate fluorescence probe, we measured the amount of ROS in cancer cells that were treated with cymantrene derivatives. The results show that production of ROS by cymantrene compounds in SKOV-3 cells is concentration and time dependent (Figure 8). The greatest increase in ROS was observed after 30 min treatment, with the highest concentration of cymantrene conjugates employed (2 × IC_50_) (Figure 8). Generally, the most cytotoxic compounds **1**, **3**, **5**, and **C** (Table 1) generated the largest amounts of ROS, although there was no direct correlation between the IC_50_ concentration of a particular compound and the produced intracellular ROS. For example, the most toxic compound for SKOV-3 cells, derivative C (IC_50_ = 7.11 µM), generated a similar amount of ROS as the 10-fold less toxic compound **1** (IC_50_ = 75.14 µM). As compounds **1**, **3**, **5**, and **C** were effective in the production ROS, as well as in the induction of apoptosis and autophagy, these processes might contribute together to the cytotoxic action of the molecules [52,53,54].

### 3.3. Evaluation of Antitrypanosomal Activity

The antitrypanocidal activity of cymamtrene derivatives **1**–**6** was determined with bloodstream forms of the *T. brucei* variant 427-221a [55], while the general cytotoxicity of the compounds was evaluated with human myeloid leukemia HL-60 cells [56], using the resazurin viability assay described previously [57]. As we recently have shown that cymantrene-uracil derivatives inhibit the growth of trypanosomes [14], we were now interested whether cymantrene conjugates containing 5-fluorouracil as nucleobase would display improved antitrypanosomal activity. The rationale behind this approach was that 5-fluorouracil is a chemotherapeutic drug and that antitumor agents are usually also active against trypanosomes [58]. Indeed, the cymantrene-5-fluorouracil derivative **1** exhibited better trypanocidal activity, but also some enhanced cytotoxicity (Table 2) when compared to its non-fluorinated counterpart tested previously (*T. brucei*: MIC = 100 µM, GI_50_ = 3.6 µM; HL-60: MIC > 100 µM, GI_50_ > 100 µM [14]). Hence, the selectivity (ratio of cytotoxic to trypanocidal activity) of compound **1** was not improved. The corresponding alcohol **2** of compound **1** was nontoxic to both trypanosomes and HL-60 cells (Table 2).

To increase the lipophilicity of the alcohol **2**, the compound was further modified to yield the triphenylphosphine derivative **3** and the methylated derivative **4**. Whereas, **3** displayed similar antitrypanocidal and cytotoxic activities as compound **1**, derivative **4** exhibited only fractionally increased antitrypanosomal activity and no cytotoxic effect (Table 2). Since modification of compound **2** with either a triphenylphosphine ligand or methyl group resulted in derivatives with increased trypanocidal activities, we were wondering whether this strategy would also help to improve the antitrypanosomal activity of the cymantrene-adenine alcohol **B**. Derivative **B** was previously found not to be a very active compound (*T. brucei*: MIC > 100 µM, GI_50_ = 30.8 µM; HL-60: MIC > 100 µM, GI_50_ > 100 µM [14]). As expected, compounds **5** (triphenylphosphine derivative of **B**) showed increased activity, while **6** (methylated derivative of **B**) displayed similar antitrypanocidal and cytotoxic activities as compound **4** (Table 2). The most active compounds **1**, **3**, **5**, and **C** were found to display trypanocidal activity that were within the range of difluoromethylornithine (DFMO) and suramin (Table 2), two drugs that are currently used to treat sleeping sickness. Although their MIC and GI_50_ ratios of cytotoxic to trypanocidal activities were only in a moderate range of 7–13, one should bear in mind that a cancer cell line was used for determining the selectivity. Therefore, when compared with non-malignant cells, it is likely that the cytoxocity of the compounds are overestimated.

In general, the results showed that lipophilic phosphine ligands increase the trypanocidal activity of the investigated compounds to a greater extent than methylation.

### 3.4. Evaluation of Antibacterial Activity

Organometallic compounds play an increasingly important role in the search for new antibacterial agents [9,10,34]. We, therefore, thought to investigate also the bactericidal activity of the newly synthesized cymantrenes **1**–**7**, compound **C** and **CymH**. The antibacterial activity of the compounds was tested against Gram-positive methicillin-sensitive *Staphylococcus aureus* (MSSA), methicillin-resistant *S. aureus* (MRSA), vancomycin-intermediate *S. aureus* (VISA), *S. epidermidis*, and Gram-negative *Escherichia coli* bacterial strains, and expressed as minimal inhibitory concentration (MIC) values (Table 3).

The nucleobase-free compounds **7** and **CymH** were not active, whereas the derivatives **1** and **C** exhibited remarkable potency against all of the Gram-positive bacterial strains, including MRSA and VISA pathogens. None of the tested compounds showed any activity against the Gram-negative *E. coli* strain. This lack of activity against *E. coli* can be probably explained by an impeded cellular uptake of the compounds through the Gram-negative cell wall that consists of two layers and effective protein efflux pump systems [60]. The antibacterial activities of **1** and **C** were further tested against four clinical isolates of *S. aureus* and two clinical isolates of *S. epidermidis* (Table 4).

Promisingly, the clinical isolates showed similar sensitivities for the two compounds as the reference strains. However, the antibacterial activity of **1** and **C** cannot be simply explained on the basis of their lipophilicity since their log *P_o/w_* values differ by three logs.

**Figure 9:**
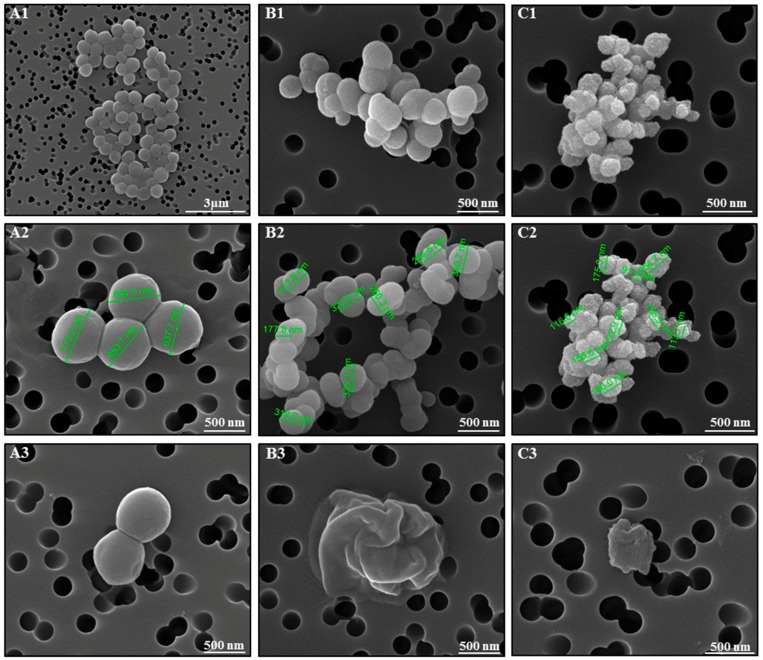
*S. aureus* morphology observed by SEM after incubation in growth medium in the absence (**A1**–**A3**, control) and presence of compound **1** (**B1**–**B3**, 48 µg/mL; **C1**–**C3**, 64 µg/mL) for 24 h.

In order to elucidate the bactericidal mechanism of action of cymantrenes, the effect of compound **1** on the morphology of *S. aureus* ATCC 25923 was exemplary studied by scanning electron microscopy (SEM) (Figure 9). Whereas, control samples exhibited the normal cellular morphology with smooth cell surfaces (Figure 9(A1–A3)), bacteria after exposure to compound **1** at concentrations of 48 µg/mL and 64 µg/mL (values between the MIC (32 µg/mL) and minimal bactericidal concentration; MBC (128 µg/mL)) for 24 h showed severe morphological alterations in cell dimensions and structure, as well as the disruption of the outer envelope (Figure 9(B1–B3) and (C1–C3)). At the highest concentration tested (64 μg/mL), a complete destruction of the bacterial cell was observed in most cases and, as a result, only bacterial debris appeared in the filters (Figure 9(C3)).

## 4. Experimental Section

### 4.1. General Information

All of the preparations were carried out using standard Schlenk techniques. Chromatographic separations were carried out using silica gel 60 (Sigma-Aldrich, St. Louis, MO, USA, 230–400 mesh ASTM). Dichloromethane, trimethylamine, dimethylform amide, and tetrahydrofuran were distilled and deoxygenated prior to use. Other solvents were of reagent grade and were used without prior purification. Adenine, 5-fluorouracil, 3-chloropropionyl chloride, propionyl chloride, sodium tetrahydridoborate, aluminium chloride, and 1,2-bis(diphenylphosphino)ethane (dppe) were purchased from commercial suppliers and were used without further purification. ^1^H-NMR (600 MHz), ^13^C{H} NMR (150 MHz), and ^31^P{^1^H} NMR (243) spectra were recorded with a Bruker Avance III 600 spectrometer (Bruker, Bremen, Germany) operating at 298 K in the Fourier transform mode. Chemical shifts are reported in δ units (ppm) using residual DMSO (^1^H δ 2.50 ppm, ^13^C δ 39.70) as reference. ^31^P NMR spectra were recorded relative to external 85% phosphoric acid standard for referencing the δ scale. Infrared spectra were recorded with an FTIR Nexus Nicolet apparatus (Nicolet, Madison, WI, USA). Mass spectra were recorded with a Varian 500-MS iT mass spectrometer (ESI) or with a Finnigan Mat95 mass spectrometer (EI). Microanalyses were determined by Analytical Services of the Polish Academy of the Sciences, Łódź.

### 4.2. Chemistry

*Synthesis of Ketone* **1**. To a stirred solution of 3-chloropropionylcymantrene (294 mg, 1.0 mmol) in DMF (20 mL), trimethylamine (278 μL 2.0 mmol) was added in a single portion at ambient temperature. After 20 min of stirring, 5-fluorouracil (130 mg, 1.0 mmol) was added and the mixture was stirred at a temperature of 75 °C for 5 h. Subsequently, the solvent was evaporated and the residue subjected to a preliminary column chromatography on SiO_2_ (eluent chloroform:methanol 50:2 (*v*/*v*)), which afforded **1**. Crude **1** was crystallized from a chloroform-*n*-pentane mixture affording the analytically pure **1**.

**1** Yellow solid, 67% (260 mg). ^1^H-NMR (600 MHz, DMSO-*d*_6_): δ = 11.74 (s, 1H, NH), 8.00 (d, *J*_H,F_ = 6.6 Hz, 1H, H-6 5-fluorouracil), 5.81 (pt, *J*_H,H_ = 2.4 Hz, 2H, Cp), 5.21 (pt, *J*_H,H_ = 2.4 Hz, 2H, Cp), 3.90 (t, *J*_H,H_ = 6.6 Hz, 2H, CH_2_), 3.08 (t, *J*_H,H_ = 6.6 Hz, 2H, CH_2_). ^13^C-NMR (150 MHz, DMSO-*d*_6_): δ = 223.4, 195.7, 157.4 (d, *J*_C,F_ = 26 Hz), 149.4, 139.2 (d, *J*_C,F_ = 228 Hz), 130.7 (d, *J*_C,F_ = 33 Hz), 91.6, 87.5, 85.3, 43.4, 37.2. MS (EI, 70 eV): *m*/*z* = 304 (M^+^ − 3CO). FTIR (KBr): 3059 (CH), 2028 (CO), 1935 (CO), 1928 (CO), 1696 (C=O), 1680 (C=O), 1663 (C=O) cm^−1^. Anal. Calcd for C_15_H_10_N_2_O_6_FMn: C, 46.41%; H, 2.60%. Found: C, 46.22%; H, 2.78%.

*Synthesis of Alcohol* **2**. To a stirred THF solution (20 mL) containing **1** (194 mg, 0.5 mmol), NaBH_4_ (19 mg, 0.5 mmol) was added in a single portion at room temperature. After 20 min of stirring, the reaction mixture was poured into 20 mL of water, extracted with chloroform, dried over MgSO_4_, and the obtained solution was evaporated to dryness. The residue was subjected to column chromatography on SiO_2_ (eluent chloroform:methanol 50:3 (*v*/*v*)). Crystallization from dichloromethane-*n*-pentane afforded the analytically pure alcohol **2**.

**2** Yellow solid, 85% (165 mg).^1^H-NMR (600 MHz, DMSO-*d*_6_): δ = 11.70 (s, 1H, NH), 8.01 (d, *J*_H,F_ = 7.2 Hz, 1H, H-6 5-fluorouracil), 5.43 (d, *J*_H,H_ = 5.4 Hz, 1H, OH), 5.08 (m, 1H, Cp), 5.06 (m, 1H, Cp), 4.89 (m, 1H, Cp), 4.87 (m, 1H, Cp), 4.24 (m, 1H, CH), 3.82 (m, 1H, CH_2_), 3.67 (m, 1H, CH_2_), 1.92 (m, 1H, CH_2_), 1.78 (m, 1H, CH_2_). ^13^C-NMR (150 MHz, DMSO-*d*_6_): δ = 225.7, 157.6 (d, *J*_C,F_ = 27 Hz), 149.6, 139.6 (d, *J*_C,F_ = 228 Hz), 130.5 (d, *J*_C,F_ = 33 Hz), 111.0, 83.1, 82.5, 82.2, 81.7, 63.9, 45.4, 36.8. MS (EI, 70 eV): *m*/*z* = 306 (M^+^ − 3CO), 288 (M^+^ − 3CO − H_2_O). FTIR (KBr): ≈3500 (OH), 3167, 3050, 2835, 2015 (CO), 1932 (CO), 1682 (C=O), 1660 (C=O) cm^−1^. Anal. Calcd for C_15_H_12_N_2_O_6_FMn: C, 46.17%; H, 3.10%. Found: C, 46.52%; H, 3.33%.

*General Procedure for the Synthesis of Compounds* **3** *and* **5**. An anhydrous THF (40 mL) solution containing either alcohol **2** (195 mg, 0.5 mmol) or **B** (198 mg, 0.5) and triphenylphosphine (131 mg, 0.5 mmol) was photolysed with a 200 W high-pressure mercury lamp for 2 h. The solvent was evaporated to dryness. The residue was subjected to column chromatography on SiO_2_ (eluent chloroform:methanol 50:1 (*v*/*v*)). Crystallization from chloroform/*n*-pentane gave the analytically pure triphenylphosphine derivatives **3** and **5**.

**3** Green solid, 47% (147 mg). ^1^H-NMR (600 MHz, DMSO-*d*_6_): δ = 11.68 (s, 1H, NH), 7.98 (d, *J*_H,F_ = 6.6 Hz, 1H, H-6 5-fluorouracil), 7.449 and 7.439 (two overlapped s, 9H, Ph), 7.36 (m, 6H, Ph), 5.20 (d, *J*_H,H_ = 6.0 Hz, 1H, OH), 4.56 (pq, *J*_H,H_ = 3.0 Hz, *J*_H,H_ = 1.8 Hz, 2H, Cp), 4.22 (m, 1H, CH), 4.02 (pd, *J*_H,H_ = 1.8 Hz, 2H, Cp), 3.83 (m, 1H, CH_2_), 3.68 (m, 1H, CH_2_), 1.96 (m, 1H, CH_2_), 1.82 (m, 1H, CH_2_). ^13^C-NMR (150 MHz, DMSO-*d*_6_): δ = 232.8 (d, *J*_P,C_ = 21 Hz), 232.6 (d, *J*_P,C_ = 18 Hz), 157.6, 149.5, 137.6 (d, *J*_P,C_ = 40 Hz), 132.4 (d, *J*_P,C_ = 10 Hz), 129.7, 128.4 (d, *J*_P,C_ = 9 Hz), 106.4, 84.1, 82.6, 81.0, 79.6, 64.3, 45.5, 35.7. ^31^P{^1^H} NMR (243 MHz, DMSO-*d*_6_): δ=91.58. MS (EI, 70 eV): *m*/*z* = 624 (M^+^), 550 (M^+^ − 2CO − H_2_O). FTIR (KBr): ≈3500 (OH), 3072, 3053, 2961, 2923, 1926 (CO), 1856 (CO), 1692 (C=O), 1660 (C=O), 1477, 1429 cm^−1^. Anal. Calcd for C_32_H_27_N_2_O_5_FPMn + MeOH: C, 60.37%; H, 4.76%. Found: C, 60.46%; H, 4.71%.

**5** Green solid, 43% (135 mg). ^1^H-NMR (600 MHz, DMSO-*d*_6_): δ = 8.12 (s, 1H, H-2 adenine), 8.09 (s, 1H, H-8 adenine), 4.429 and 7.421(two overlapped s, 9H, Ph),7.34 (m, 6H, Ph), 7.13 (s, 2H, NH_2_), 5.34 (d, *J*_H,H_ = 6.0 Hz, 1H, OH), 4.56 (pt, *J*_H,H_ = 1.8 Hz, 2H, Cp), 4.26 (m, 2H, CH_2_), 4.16 (m, 1H, CH), 4.00 (bs, 2H, Cp), 2.17 (m, 1H, CH_2_), 2.01 (m, 1H, CH_2_). ^13^C-NMR (150 MHz, DMSO-*d*_6_): δ = 228.0, 164.0, 153.7, 153.0, 150.0, 137.1 (d, *J*_P,C_ = 40 Hz), 132.8 (d, *J*_P,C_ = 20 Hz), 131.9 (d, *J*_P,C_ = 11 Hz), 129.2, 128.6, 128.4 (d, *J*_P,C_ = 8 Hz), 127.9 (d, *J*_P,C_ = 11 Hz), 106.0, 95.8, 83.2, 82.5, 80.5, 79.3, 63.8, 37.1. ^31^P{^1^H} NMR (243 MHz, DMSO-*d*_6_): δ = 91.67. MS (EI, 70 eV): *m*/*z* = 629 (M^+^), 555(M^+^ − 2CO − H_2_O). FTIR (KBr): ≈3500 (OH), 2958, 2927, 1929 (CO), 1860 (CO), 1635 (NH), 1591 (NH) cm^−1^. Anal. Calcd for C_33_H_29_N_5_O_3_PMn: C, 62.96%; H, 4.64%; N, 11.12%. Found: C, 62.71%; H, 4.88%; N, 11.04%.

*Synthesis of Compound* **4**. Solid NaH (24 mg, 1.0 mmol) was added to a stirred solution of alcohol **2** (195 mg, 0.5 mmol) in DMF (20 mL) at ambient temperature. After stirring for 5 min, methyl iodide (62 μL, 142 mg, 1.0 mmol) was added in one portion at ambient temperature. After 20 min of stirring, the reaction mixture was poured into 25 mL of water, extracted with chloroform, dried over MgSO_4_, and the obtained solution was evaporated to dryness. The residue was subjected to column chromatography on SiO_2_ (eluent chloroform). Chromatography gave the analytically pure product **4**.

**4** Yellow oil, 40% (84 mg). ^1^H-NMR (600 MHz, DMSO-*d*_6_): δ = 8.12 (d, *J*_H,F_ = 6.0 Hz, 1H, H-6 5-fluorouracil), 5.18 (s, 1H, Cp), 5.12 (s, 1H, Cp), 4.95 (s, 1H, Cp), 4.91 (s, 1H, Cp), 3.92 (t, *J*_H,H_ = 6.0 Hz, 1H, CH), 3.80 (q, *J*_H,H_ = 6.0 Hz, 2H, CH_2_), 3.17 (s, 3H, CH_3_), 2.00 (q, *J*_H,H_ = 6.0 Hz, 2H, CH_2_). ^1^H-NMR (600 MHz, after D_2_O addition, DMSO-*d*_6_ ): δ = 8.10 (bs, 1H, H-6 5-fluorouracil), 5.16 (s, 1H, Cp), 5.11 (s, 1H, Cp), 4.94 (s, 1H, Cp), 4.90 (s, 1H, Cp), 3.91 (s, 1H, CH), .79 (bs, 2H, CH_2_), 3.28 (s, 3H, OCH_3_), 3.16 (s, 3H, NCH_3_), 1.98 (bs, 2H, CH_2_). ^13^C-NMR (150 MHz, DMSO-*d*_6_): δ = 224.9, 156.5 (d, *J*_C,F_ = 25 Hz), 149.3, 138.7 (d, *J*_C,F_ = 225 Hz), 128.4 (d, *J*_C,F_ = 34 Hz), 104.8, 83.7, 83.2, 82.1, 81.6, 73.5, 56.1, 45.6, 33.6, 27.6. MS (EI, 70 eV): *m*/*z* = 418 (M^+^), 334 (M^+^ − 3CO), 302 (M^+^ − 3CO − CH_3_OH). FTIR (KBr): 2021 (CO), 1923 (CO), 1711 (C=O), 1679 (C=O), 1651 (C=O) cm^−1^. Anal. Calcd for C_17_H_16_N_2_O_6_FMn + H_2_O: C, 46.80%; H, 4.16%. Found: C, 46.86%; H, 4.25%.

*Synthesis of Compound* **6**. Solid NaH (36 mg, 1.5 mmol) was added to a stirred solution of alcohol **B** (198 mg, 0.5 mmol) in DMF (20 mL) at ambient temperature. After stirring for 2 h, methyl iodide (93 μL, 213 mg, 1.5 mmol) was added in one portion at ambient temperature. After 4 h of stirring, the reaction mixture was poured into 25 mL of water, extracted with chloroform, dried over MgSO_4_, and the obtained solution was evaporated to dryness. The residue was subjected to column chromatography on SiO_2_ (eluent chloroform). Crystallization from chloroform/*n*-pentane gave the analytically pure compound **6**.

**6** Colorless solid, 79% (173 mg). ^1^H-NMR (600 MHz, DMSO-*d*_6_): δ = 8.20 (s, 1H, H-2 adenine), 8.13 (s, 1H, H-8 adenine), 5.22 (m, 1H, Cp), 5.13 (m, 1H, Cp), 4.93 (pq, *J*_H,H_ = 2.4 Hz, *J*_H,H_ = 1.8 Hz, 1H, Cp), 4.90 (pq, *J*_H,H_ = 2.4 Hz, *J*_H,H_ = 1.8 Hz, 1H, Cp), 4.25 (m, 2H, CH_2_), 3.80 (dd, *J*_H,H_ = 8.7 Hz, *J*_H,H_ = 3.6 Hz, 1H, CH), 3.44 (bs, 6H, N(CH_3_)_2_), 2.21 (m, 2H, CH_2_). ^1^H-NMR (600 MHz, after D_2_O addition, DMSO-*d*_6_): δ = 8.18 (s, 1H, H-2 adenine), 8.11 (s, 1H, H-8 adenine), 5.18 (s, 1H, Cp), 5.09 (s, 1H, Cp), 4.90 (s, 1H, Cp), 4.87 (s, 1H, Cp), 4.24 (m, 2H, CH_2_), 3.77 (dd, *J*_H,H_ = 8.4 Hz, *J*_H,H_ = 3.6 Hz, 1H, CH), 3.50 (bs, 6H, N(CH_3_)_2_ adenine), 3.27 (s, 3H, OCH_3_), 2.18 (m, 2H, CH_2_). ^13^C-NMR (150 MHz, DMSO-*d*_6_): δ = 225.3, 154.3, 151.7, 150.4, 139.7, 119.3, 104.8, 84.3, 83.8, 82.4, 81.9, 73.7, 56.6, 35.3. MS (EI, 70 eV): *m*/*z* = 437 (M^+^), 422 (M^+^ − CH_3_), 353 (M^+^ − 3CO) FTIR (KBr): 3072 (CH), 3037 (CH), 2927 (CH), 2819 (CH), 2018 (CO), 1929 (CO), 1600, 1568 cm^−1^. Anal. Calcd for C_19_H_20_N_5_O_4_Mn: C, 52.18%; H, 4.61%; N, 16.01%. Found: C, 52.23%; H, 4.68%; N, 16.19%.

*Synthesis of Compound* **7**. To a stirred solution of cymantrene (204 mg, 1.0 mmol) in dichloromethane (20 mL), propionylchloride (88 μL, 1 mmol) and AlCl_3_ (133 mg, 1.0 mmol) was added. After 4 h at ambient temperature, the reaction mixture was poured into a 3% aqueous HCl solution. Resulting dichloromethane and water phases were separated. The dichloromethane phase was dried over MgSO_4_ and evaporated to dryness. The residue was dissolved in dichloromethane and was subjected to column chromatography on SiO_2_ (eluent dichloromethane) to give compound **7**. Crystallization from dichloromethane/n-pentane afforded the analytically pure **7**.

**7** Yellow solid, 71% (184 mg). ^1^H-NMR (600 MHz, DMSO-*d*_6_): δ = 5.77 (pt, *J*_H,H_ = 2.4 Hz, 2H, Cp), 5.19 (pt, *J*_H,H_ = 2.4 Hz, 2H, Cp), 2.66 (q, *J*_H,H_ = 7.2 Hz, 2H, CH_2_), 1.02 (t, *J*_H,H_ = 7.2 Hz, 3H, CH_3_). ^13^C-NMR (150 MHz, DMSO-*d*_6_): δ = 223.8, 198.3, 92.2, 87.4, 85.2, 31.8, 8.1. MS (EI, 70 eV): *m*/*z* = 260 (M^+^). FTIR (KBr): 3091 (CH), 2987 (CH), 2842 (CH), 2908 (CH), 2027 (CO), 1942 (CO), 1676 (C=O) cm^−1^. Anal. Calcd for C_11_H_9_O_4_Mn: C, 50.79%; H, 3.49%. Found: C, 50.90%; H, 3.64%.

### 4.3. Single-Crystal X-ray Structure Analysis of ***1***, ***6*** and ***C***

Good quality single-crystals of **1**, **6** and **C** were selected for the X-ray diffraction experiments at *T* = 100(2) K. Diffraction data were collected on the Agilent Technologies SuperNova Dual Source diffractometer (Yarnton, UK) with Cu*K*α radiation (*λ* = 1.54184 Å) using CrysAlis RED software (CrysAlis CCD and CrysAlis RED, Oxford Diffraction, Oxford Diffraction Ltd.: Yarnton, UK, 2008) [61]. In all cases, the analytical numerical absorption correction was applied [61] using a multifaceted crystal model based on expressions that were derived by Clark and Reid (1994) [62], as implemented in the SCALE3 ABSPACK scaling algoritm [61]. The structural determination procedure was carried out using the SHELX package [63]. The structures were solved by direct methods and then successive least-square refinement was carried out based on the full-matrix least-squares method on *F*^2^ using the SHELXL program [63]. All of the H-atoms bound to C-atoms were positioned geometrically, with C–H equal to 0.93, 0.96, 0.97 and 0.98 Å for aromatic, methyl, methylene and methine H-atoms, respectively, and were constrained to ride on their parent atoms with *U*_iso_(H) = x*U*_eq_(C), where x = 1.2 for the aromatic, methylene and H-atoms, and x = 1.5 for the methyl H-atoms. The H-atoms that were linked to N-atoms were located on a Fourier difference map and refined as riding with *U*_iso_(H) = 1.2*U*_eq_(N). In the case of **1**, a few distinct peaks on the difference Fourier map were indicating the presence of a disordered solvent molecule. However, all of the attempts to model a disordered solvents used for crystallization failed. Therefore, the solvent contribution was removed by applying the appropriate MASK procedure in the Olex2 program [64]. Calculated void volume was approximately 204.7 Å^3^ occupied by 48.4 electrons per unit cell. The figures presented in this work were prepared using Olex2 and ORTEP-3 programs [64,65]. The molecular interactions were identified using PLATON [66].

### 4.4. Determination of the Log P_o/w_

For the determination of log *P_o/w_* values, the newly synthesized compounds were analyzed by an Agilent 1200 system (Agilent Technologies, Santa Clara, CA, USA) equipped with a Zorbax Eclipse XDB column (C18, 250 × 4.6 mm, Agilent Technologies) and controlled by the software ChemStation Data Analysis. The separation was isocratic, and water/methanol (HPLC gradient grade, Sigma-Aldrich, St. Louis, MO, USA) mixtures containing 0.50, 0.55, 0.60, 0.65, 0.70, 0.75, 0.80, 0.85, 0.90 and 0.95 volume fractions of organic modifier were used as the mobile phase. The flow rate was 1.0 mL/min for the **7** and **2** and 0.8 mL/min for other compounds. The chromatographic peaks of test compounds were detected by UV absorbance at 230 nm. The column temperature was kept at 20 °C. The dead-time values were measured from the effluent peaks. All of the runs were performed in triplicate, and the resulting average values were used for the calculation of the retention factors.

### 4.5. Biology

#### 4.5.1. Anticancer Activity

##### Cell Lines

Anticancer activity of cymantrene derivetives were determined on the basis of their cytotoxic effects on a panel of human cancer cells, as derived from different types of solid tumors: estrogen responsive breast adenocarcinoma (MCF-7), triple negative breast adenocarcinoma (MDA-MB-231), ovarian adenocarcinoma (SKOV-3), non-small cell lung carcinoma (A549), hepatocellular carcinoma (HepG2), and glioblastoma (U-87 MG). Additionally, doxorubicin resistant breast adenocarcinoma cells (MCF-7/Dox) were also included for comparison. All of the cell lines were purchased from American Type Culture Collection (ATCC, Rockville, MD, USA), except the MCF-7/dox cell line, which was a generous gift from Professor Malgorzata Latocha from the Department of Cell Biology, Medical University of Silesia in Katowice, Poland.

##### Cell Culture

Cells were grown as a monolayer in DMEM growth medium, supplemented with 10% FBS, penicillin (10 U/mL) and streptomycin (50 μg/mL), under standard conditions (37 °C, 5% CO_2_ and 95% air, 100% humidity) and periodically checked for *Mycoplasma* contamination.

##### Cytotoxicity Assay

The cytotoxicity of cymantrene derivatives was evaluated by the MTT microplate methods as described elsewhere [67] by incubating the cells with increasing concentrations of tested compounds for 24 h. Briefly, 0.1 mL of logarithmically growing cells were seeded in a 96-well microplate (5 × 10^4^ cells/well) a day before the experiments and after 24 h of culture, tested compounds, dissolved in DMSO, and diluted in culture medium, were added. The final concentration of DMSO did not exceed 0.1%. After further incubation for 24 h, the medium was replaced with fresh medium and the cells were allowed to grow for additional 48 h. Then, the medium was aspirated and 0.05 mL of MTT (final concentration of 5 mg/mL medium) added to each well, and the cells were incubated for further 4 h. The absorbance of the product, resulting from the MTT reduction, was read with a microplate reader at 545 nm. The percentage of viable cells was calculated by comparing the absorbance of treated cells with that of the untreated control cells (taken as 100%). The IC_50_ concentration of each compound was calculated from the cell survival curves using GraphPad Prism 4.02 software for Windows (GraphPad Software, San Diego, CA, USA).

##### Determination of Autophagy and Apoptotic/Necrotic Cell Death

One day prior to the experiments, cells were seeded at the appropriate density into 35 mm diameter dishes. The next day, test compounds were added at three different concentrations: 0.5 × IC_50_, 1 × IC_50_, and 2 × IC_50_ (the final concentration of DMSO in the medium did not exceed 0.1%) and the cells were incubated with the compounds for 24 h. At the end of incubation, the medium was aspirated, the cell monolayer was washed twice with pre-warmed PBS, and fresh medium was added. The cells were cultured in drug-free medium for an additional 24 or 48 h to enable them to recover after the exposure to the compounds and to repair any induced damages.

Quantitative analysis of morphological changes that are associated with apoptosis, necrosis, and autophagy were assessed using a fluorescence microscope (Olympus IX70, Tokyo, Japan) at 400× magnification, as described elsewhere [67]. At the indicated time points, cells were washed with PBS, trypsinized, and centrifuged at 1000 rpm (fixed angle rotor, 72× *g*) for 5 min at 37 °C. The supernatant was removed and the cell pellet (100 µL) was transferred to the Eppendorf tube, mixed with Ho33258 (2 µL, 0.13 mM) and PI (2 µL, 0.23 mM), and incubated for 5 min in the dark at room temperature. At least 300 stained cells were counted in triplicate in each of the three independent experiments. Cells were classified as live, apoptotic, or necrotic on the basis of their morphological and staining characteristics, and each fraction was related to the total cell number. Double staining with these fluorescence dyes enables the identification of the status of any cell: live cells with uniformly pale-blue fluorescent nuclei; early apoptotic cells with intact membranes but condensed and fragmented chromatin giving rise to an intense bright-blue fluorescence of the nuclei; late-apoptotic cells with blue-violet fluorescence due to condensed and fragmented chromatin and permeabilized plasma membranes allowing for small amounts of PI to enter the cells; and, swollen necrotic cells with damaged plasma membranes giving rise to red fluorescence caused by PI.

The ability of tested compounds to induce autophagosome formation inside cells (the process associated with the induction of autophagy) was evaluated by microscopic examination of the morphology of cells stained with acridine orange (AO).

##### Determination of Intracellular ROS

The intracellular ROS were measured with the oxidation-sensitive fluorescent probes 2′,7′-dichlorodihydrofluorescein diacetate (H_2_DCF-DA) (Sigma-Aldrich, St. Louis, MO, USA), as described elsewhere [68]. The cell-permeant non-fluorescent dye H_2_DCF-DA is a reduced form of fluorescein that inside the cell, upon cleavage of the acetate groups by intracellular esterases and oxidation by ROS, is converted into the highly fluorescent product 2′,7′-dichlorofluorescein (DCF) (λ_ex_/λ_em_: 498/525 nm). For the assay, 2 × 10^4^ cells were seeded into wells of black flat-bottom 96-well microplates a day before the experiment. The next day, test compounds were added at concentrations of 0.5 × IC_50_, 1 × IC_50_, and 2 × IC_50_. The final concentration of DMSO did not exceed 0.1%. After 24 h incubation, medium was removed, cell monolayers were washed twice with HBSS and 5 µM H_2_DCF-DA, dissolved in HBSS, was added to each well. The cells were incubated with the fluorescent probe for 30 min in the dark at 37 °C, and then the fluorescence of produced DCF was monitored over a time period of 0–3 h at 15-min intervals with a microplate reader.

#### 4.5.2. Statistical Analysis

All data are expressed as a mean ± SD. An ANOVA analysis of variance with a Tuckey post hoc test were used for multiple comparisons. A statistical program STATISTICA (StatSoft, Tulsa, OK, USA) was used for all statistical calculations. A *p* value of <0.05 was considered significant.

#### 4.5.3. Trypanocidal Assay

Bloodstream forms of *T. brucei* clone 427-221a [69] and human myeloid leukemia HL-60 cells [56] were grown in Baltz medium [70] and were supplemented with 16.7% heat-inactivated fetal calf serum. All of the cultures were maintained at 37 °C in a humidified atmosphere containing 5% of CO_2_. Toxicity assays were performed, as previously described, with some modifications [71]. In brief, trypanosomes and HL-60 cells were seeded at an initial cell density of 10^4^/mL and 5 × 10^4^/mL, respectively, in 96-well plates in a final volume of 200 μL culture medium containing various concentrations of test compounds dissolved in 100% DMSO. It should be noted that none of the test compounds formed precipitates when diluted with medium. The controls contained DMSO alone. In all of the experiments, the final DMSO concentration was 0.9%. After 24 h incubation, 20 µL of a 0.5 mM resazurin solution prepared in PBS was added and the cultures were incubated for a further 48 h. Resazurin is a vital dye that, when being reduced by living cells, changes its colour from blue to pink. Thus, by measuring absorbance, the proliferation of cells can be easily determined. The change in absorbance was read with a microplate reader using a test wavelength of 570 nm and a reference wavelength of 630 nm. GI_50_ values were calculated by linear interpolation according to the method described by Huber and Koella [72]. MIC values were determined microscopically.

#### 4.5.4. Microbiology

The antibacterial activity of **CymH**, **1**–**7** and **C** and the reference drugs penicillin G and polymyxin B was tested by the liquid microdilution method under standard conditions using twofold dilution series, as described in the Clinical and Laboratory Standards Institute (CLSI), reference method M07-A8, and expressed as minimal inhibitory concentration (MIC) values [73]. A panel of Gram-positive bacterial strains, *S. aureus* ATCC^®^ 29213 (sensitive to methicillin, (MSSA)), *S. aureus* ATCC^®^ 43300 (resistant to methicillin, (MRSA)), *S. aureus* ATCC 700787™ (intermediate to vancomycin, (VISA)), and *S. epidermidis* ATCC 12228™, and one Gram-negative, strain *E. coli* ATCC BAA-198™ (ESBL) were used. In addition, four clinical isolates of *S. aureus* (MSSA and MRSA) and two clinical isolates of *S. epidermidis* that were collected from different patients at the Medical University of Warsaw, Poland, were also tested. The bacterial cells were cultivated on tryptic soy agar (TSA) and nutrient agar (NA), according to ATCC recommendations. All of the strains were incubated at 37 °C. Tested compounds were dissolved in DMSO and then serially diluted twofold with cation-adjusted Mueller-Hinton broth (CAMHB). Then, 95 µL of the dilutions were dispensed into microdilution sterile plates (Mar-Four), followed by the addition of 5 µL of bacteria inoculum containing 5 × 10^4^ CFU/mL. The final concentrations of the compounds ranged from 512 to 2 µg/mL. Each compound was tested in triplicate, and Penicillin G (8–0.15 µg/mL) and Polymyxin B (256–0.0625 µg/mL) were used as controls. The plates were incubated at 37 °C for 18 to 24 h depending on the bacterial strain. Thereafter, the absorbance was measured at *λ* = 540 nm and *λ* = 595 nm using a Spectrostar Omega microplate reader (BMG Labtech, Offenburg, Germany). The MIC value was defined as the lowest concentration of a compound that reduced the growth by 100%.

The effect of compound **1** on bacterial morphology was studied by SEM, as previously described [74]. Briefly, samples of *S. aureus* ATCC 25923 (10^5^ CFU/mL) were centrifuged (600× *g*, 5 min) and resuspended in growth medium containing compound **1** at concentration of 48 or 64 µg/mL. After incubation for 24 h at 37 °C, the cells were washed twice with PBS (0.1 × PBS), fixed with 2.5% glutaraldehyde (overnight), filtered (0.2 μm, pore size cutoff), and subsequently dehydrated in a series of ethanol washes (30%, 50%, 70%, 80%, 90% and 100%; twice for 15 min). The solvent was then evaporated and the samples were coated with a thin layer of platinum (15 nm). SEM images were recorded using a SEM Inspect F50 (FEI Co., Eindhoven, The Netherlands) at an energy range of 10–15 keV.

#### 4.5.5. Deposited Data

Cif files of the structure determinations were deposited at the Cambridge Crystallographic Data Center (CCDC numbers 1575005-1575007). These data can be obtained free of charge via www.ccdc.cam.ac.uk/structures.

## 5. Conclusions

This study indicates that cymantrene derivatives display biological activities against a variety of different cell types. In addition to recently published results on the trypanocidal activity of cymantrene compounds, here we show that some of these organometallics also exhibit anticancer and antibacterial activities. Evidence was provided that cytotoxic and antiproliferative properities of investigated cymantrene derivatives toward human cancer cells could stem from the abilities of these compounds to induce apoptosis, autophagy, and oxidative stress, which could be considered as possible mechanisms of anticancer activity of cymantrenes. This assumption, however, needs further more detailed studies. The most prominent feature of biological activity of investigated cymantrene derivatives is their selective anticancer activity toward cancer cells that were derived from triple negative breast adenocarcinoma (MDA-MB-231), doxorubicin-resistant hormone-dependent breast adenocarcinoma (MCF-7/DOX), ovarian adenocarcinoma (SCOV-3), and non-small lung cell adenocarcinoma (A549), cancers refractory to conventional chemotherapy.

In bacteria, cymantrenes seemed to cause shrinking of cells, although it remains uncertain whether this process was a direct effect of the cymantrene derivatives or whether the compounds that prevented the growth of the cells and the observed smaller cell size was the result of ongoing cell division. Another important finding of the study is that cymantrene compounds containing a triphenylphosphine ligand displayed generally higher biological activity.

## Figures and Tables

**Table 1:** Lipophilicity (log *P_o/w_*) and cytotoxicity (IC_50_; μM) of compounds **CymH**, **1**–**7**, **B**, and **C** against human cancer cells ^a^.

Compound	Log *P_o/w_*	SKOV-3	MCF-7	MCF-7/DX	MDA-MB-231	A549	HepG2	U87-MG
**CymH**	3.5	121 ± 3.05	86.01 ± 1.30	143.55 ± 2.24	68.60 ± 1.74	87.05 ± 1.35	74.66 ± 3.73	84.44 ± 4.22
**1**	2.6	75.14 ± 3.76	70.48 ± 2.52	137.49 ± 3.41	27.86 ± 1.11	25.05 ± 1.25	114.45 ± 2.72	64.47 ± 3.22
**2**	2.8	97.48 ± 2.87	119.45 ± 2.97	nd *	>150	**7.24 ± 0.34**	>150	100.29 ± 1.01
**3**	5.2	53.01 ± 2.65	**57.78 ± 2.89**	**39.92 ± 1.41**	35.73 ± 1.44	20.35 ± 1.09	65.76 ± 3.29	24.89 ± 1.24
**4**	3.5	>150	>150	nd	>150	>150	>150	>150
**5**	4.8	63.83 ± 3.19	102.16 ± 1.11	nd	55.95 ± 2.07	23.72 ± 0.86	106.23 ± 2.31	54.19 ± 2.71
**6**	3.5	77.1 ± 3.86	111.34 ± 3.57	nd	>150	101.51 ± 1.08	86.89 ± 2.34	130.61 ± 3.53
**7**	3.2	69.84 ± 3.49	>150	nd	63.01 ± 1.32	30.61 ± 1.35	109.57 ± 2.48	83.62 ± 1.18
**B**	2.6	>150	>150	nd	>150	>150	>150	>150
**C**	5.7	**7.11 ± 0.36**	103.74 ± 1.19	nd	43.07 ± 1.95	16.47 ± 0.82	66.59 ± 3.33	38.26 ± 1.91
**DOX**	nd	0.858 ± 0.044	**0.439 ± 0.026**	**48.50 ± 2.78**	1.073 ± 0.062	0.300 ± 0.018	0.569 ± 0.021	0.610 ± 0.029

^a^ SKOV-3 (ovarian adenocarcinoma), MCF-7 (hormone-responsive breast adenocarcinoma), MCF-7/DX (estrogen-responsive breast adenocarcinoma resistant to doxorubicin), MDA-MB-231 (triple negative breast adenocarcinoma), A549 (non-small cell lung carcinoma), HepG2 (hepatocellular carcinoma) and U87-MG (glioblastoma). The cells were incubated for 24 h with cymantrene compounds (cytotoxic effect). Results are expressed as means ± SEM of at least three independent experiments in six repeats each. In MCF-7/DX cells only the cytotoxicity of cymantrenes showing IC_50_ < 100 µM) in the parental MCF-7 cell line was estimated. nd—not determined. Cytotoxicity of cymantrene compounds showing IC_50_ > 100 µM in the parental MCF-7 cell line was not determined.

**Table 2:** The in vitro antitrypanosomal and cytotoxic activity of **CymH**, compounds **1**–**6** and the reference suramin against *T. brucei* and human myeloid leukemia HL-60 cells.

	*T. brucei*		HL-60		Selectivity	
Compound	MIC ^a^ (μM)	GI_50_^b^ (μM)	MIC (μM)	GI_50_ (μM)	MIC Ratio	GI_50_ Ratio
**CymH**	100	44.6 ± 12.3	>100	>100	>1	>2.2
**1**	10	3.02 ± 0.14	100	33.1 ± 3.4	10	11.0
**2**	>100	>100	>100	>100	1	1
**3**	10	4.27 ± 0.13	100	30.6 ± 2.6	10	7.2
**4**	100	29.6 ± 6.8	>100	>100	>1	>3.4
**5**	10	3.14 ± 0.31	100	28.7 ± 3.0	10	9.1
**6**	100	26.1 ± 1.9	>100	95.2 ± 44.4	>1	3.6
**B** ^c^	>100	30.8 ± 3.5	>100	>100	1	>3.2
**C** ^c^	10	1.67 ± 0.37	100	21.8 ± 8.6	10	13.1
**DFMO** ^d^	>125	23.9	n.d.	n.d.	n.d.	n.d.
**Suramin**	0.1–1	0.039 ± 0.003	>100	>100	>1000–100	>2564

^a^ MIC (minimum inhibitory concentration, i.e., the concentration of the compounds at which all cells were killed); ^b^ GI_50_ (50% growth inhibition, i.e., the concentration of a compound necessary to reduce the growth rate of cells by 50% to that of controls); ^c^ Data from [14]; ^d^ Data from [59]; n.d. not determined.

**Table 3:** In vitro antibacterial activity of complexes **1**–**6**, **C**, and the **7**, **CymH** compounds against reference bacterial strains (MIC μg/mL) ^a^.

Compound	MIC (μg/mL)				
	*S. aureus* ATCC^®^ 29213™ (MSSA)	*S. aureus* ATCC^®^ 43300™ (MRSA)	*S. aureus* ATCC^®^ 700787™ (VISA)	*S*. *epidermidis* ATCC^®^ 12228™	*E. coli* ATCC^®^ BAA-198™
**C**	32	32	32	16	>128
**1**	64	32	32	8	>512
**2**	>512	>512	>512	>512	>512
**3**	>256	>256	>256	>256	>256
**4**	>512	>512	>512	>512	>512
**5**	>256	>256	>256	>256	>256
**6**	>512	>512	>512	>512	>512
**7**	>512	>512	>512	>512	>512
**CymH**	>512	>512	>512	>512	>512
**penicillin G**	2	16	32	2	>256
**polymyxin B**	256	256	>256	64	0.125

^a^ MIC (the concentration of a compound necessary to reduce the growth of bacterial cells by 50% to that of controls) value of the reference compound, penicillin G. against *S. aureus* ATCC^®^ 29213 is 2 µg/mL.

**Table 4:** In vitro antibacterial activity of complexes **1** and **C** compounds against clinical isolates (MIC μg/mL) ^a^.

Compound	MIC (μg/mL)					
	Clinical *S. aureus* (MSSA) 26/11	Clinical *S. aureus* (MSSA) 30/11	Clinical *S. aureus* (MRSA) 31/12	Clinical *S. aureus* (MRSA) 44/12	Clinical *S. epidermidis* (MSSA) 37/12	Clinical *S. epidermidis* (MSSA) 43/12
**C**	32	32	32	32	16	16
**1**	32	64	64	32	4	8
**penicillin G**	0.5	2	32	8	16	8
**polymyxin B**	256	256	256	256	128	64

^a^ MIC (the concentration of a compound necessary to reduce the growth of bacterial cells by 50% to that of controls) value of the reference compound, penicillin G against *S. aureus* ATCC^®^ 29213 is 2 µg/mL.

## Supplementary Materials

Supplementary materials are available online.
