# Supplementary material for: Cymantrenyl-Nucleobases: Synthesis, Anticancer, Antitrypanosomal and Antimicrobial Activity Studies
Source: Molecules. 2017 Dec 14;22(12):2220. doi: 10.3390/molecules22122220 (PMC6149849; doi:10.3390/molecules22122220)
Supplement: Supplementary file 1 [file molecules-22-02220-s001.pdf]

Cymantrenyl-nucleobases: synthesis, anticancer, antitrypanosomal and antimicrobial activity studies

Artur Jabłoński,[a] Karolina Matczak,[b] Aneta Koceva-Chyła,[b] Kamil Durka,[b] Dietmar Steverding,[c] Katarzyna Jakubiec-Krześniak,[d] Jolanta Solecka,[d] Damian Trzybiński,[e] Krzysztof Woźniak,[e] Vanesa Andreu[f], Gracia Mendoza[f], Manuel Arruebo[f,g], Krzysztof Kochel,[b] Barbara Krawczyk,[h] Dominik Szczukocki,[h] Konrad Kowalski,[a]*

[a]Faculty of Chemistry, Department of Organic Chemistry, University of Łódź, Tamka 12, PL-91403 Łódź, Poland
[b]Department of Medical Biophysics, Faculty of Biology and Environmental Protection, University of Łódź, Pomorska 141/143, PL-90236 Łódź, Poland
[c]Bob Champion Research & Education Building, Norwich Medical School, University of East Anglia, Norwich Research Park, Norwich NR4 7UQ, United Kingdom
[d]National Institute of Public Health-National Institute of Hygiene, Chocimska 24, PL-00791 Warszawa, Poland
[e]Biological and Chemical Research Centre, Department of Chemistry, University of Warsaw, Żwirki i Wigury 101, PL-02-089 Warszawa, Poland
[f]Department of Chemical Engineering. Aragon Institute of Nanoscience (INA), University of Zaragoza, Campus Río Ebro-Edificio I+D, C/ Poeta Mariano Esquillor S/N, 50018-Zaragoza, Spain
[g]Networking Research Center on Bioengineering, Biomaterials and Nanomedicine, CIBER-BBN, 28029-Madrid, Spain
[h]Faculty of Chemistry, Department of Inorganic and Analytical Chemistry, University of Łódź, Tamka 12, 91403 Łódź, Poland

Electronic Supplementary Information

Electronic Supplementary Information

Contents

Fig. S1 ^{1}H-NMR of compound **1** **4**

Fig. S2 ^{1}H-NMR of compound **2** **5**

Fig. S3 ^{1}H-NMR of compound **3** **6**

Fig. S4 ^{1}H-NMR of compound **4** **7**

Fig. S5 ^{1}H-NMR of compound **5** **8**

Fig. S6 ^{1}H-NMR of compound **6** **9**

Fig. S7 ^{1}H-NMR of compound **7** **10**

Fig. S8 ^{31}P-NMR of compound **3** **11**

Fig. S9 ^{31}P-NMR of compound **5** **12**

Table S1. Crystallographic data and structural refinement details of **1**, **6** and chloroform solvate of **13**

Table S2. Bond lengths for **1A** **14-15**

Table S3. Valence angles for **1B** **16-18**

Table S4. Bond lengths for **6** **19**

Table S5. Valence angles for **6** **20-21**

Table S6. Bond lengths for chloroform solvate of **C** **22-23**

Table S7. Valence angles for chloroform solvate of **C** **24-26**

Fig. S10 The molecular drawing of the chloroform solvate of **C** **27**

Table S8 Equations, R^2 coefficients and log $P_{o/w}$ values **28**

Fig. S11A,B Survival curves of human SKOV-3, (MCF-7, MCF-7/DX, MDA-MB-231, A549, HepG2, U87-MG cancer cells. **29, 30**

Fig. S12A-C Induction of apoptosis and autophagy in human ovarian adenocarcinoma cells SKOV-3 **31-33**

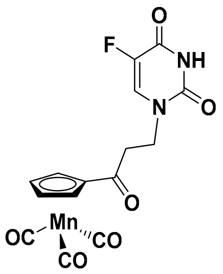

1

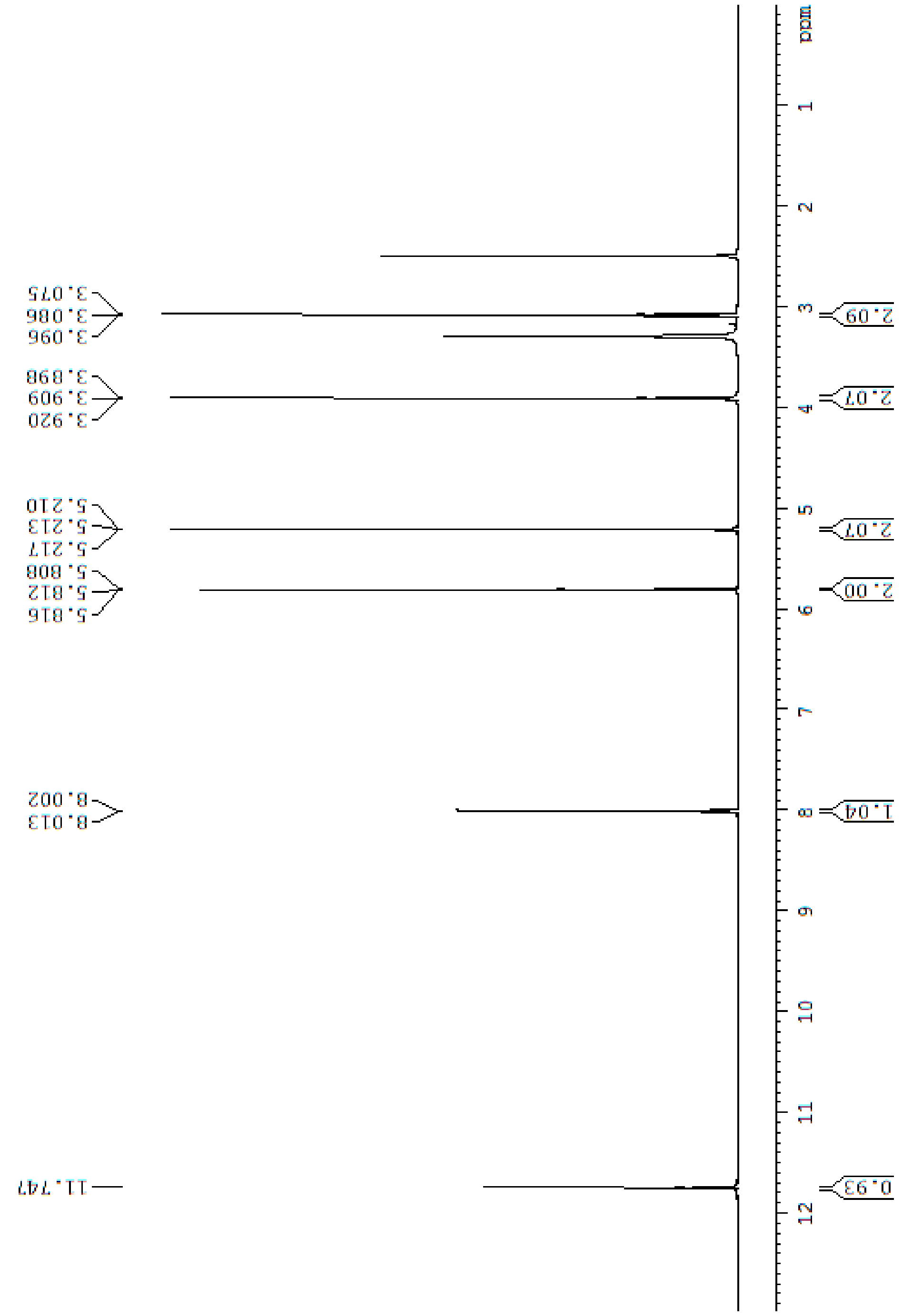

Fig. S1 ^{1}H-NMR of compound **1**.

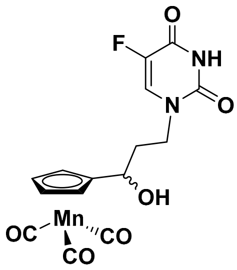

2

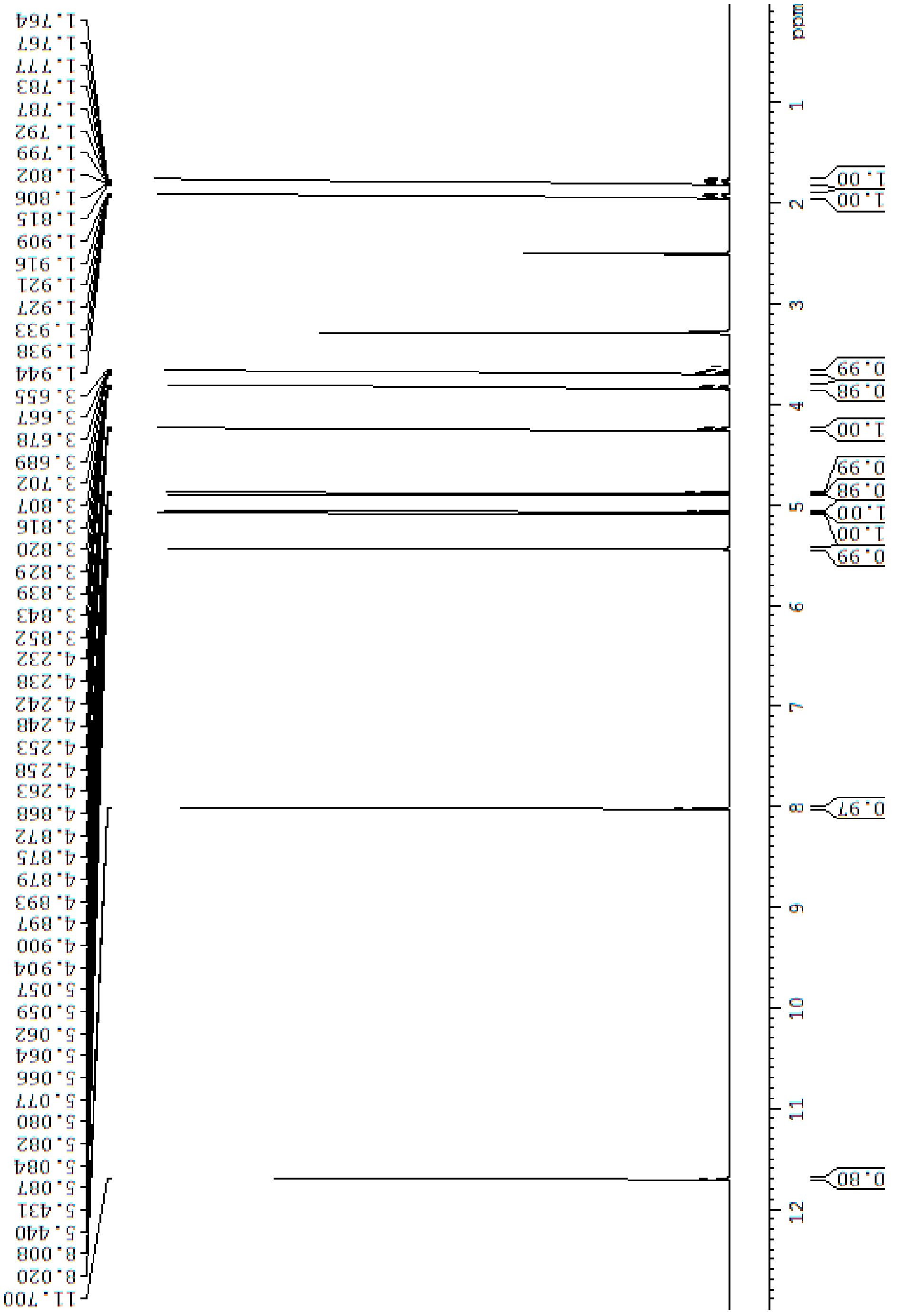

Fig. S2 ¹H-NMR of compound **2**.

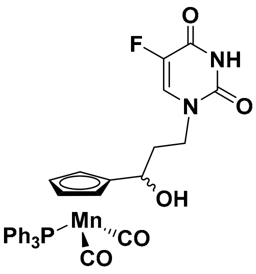

3

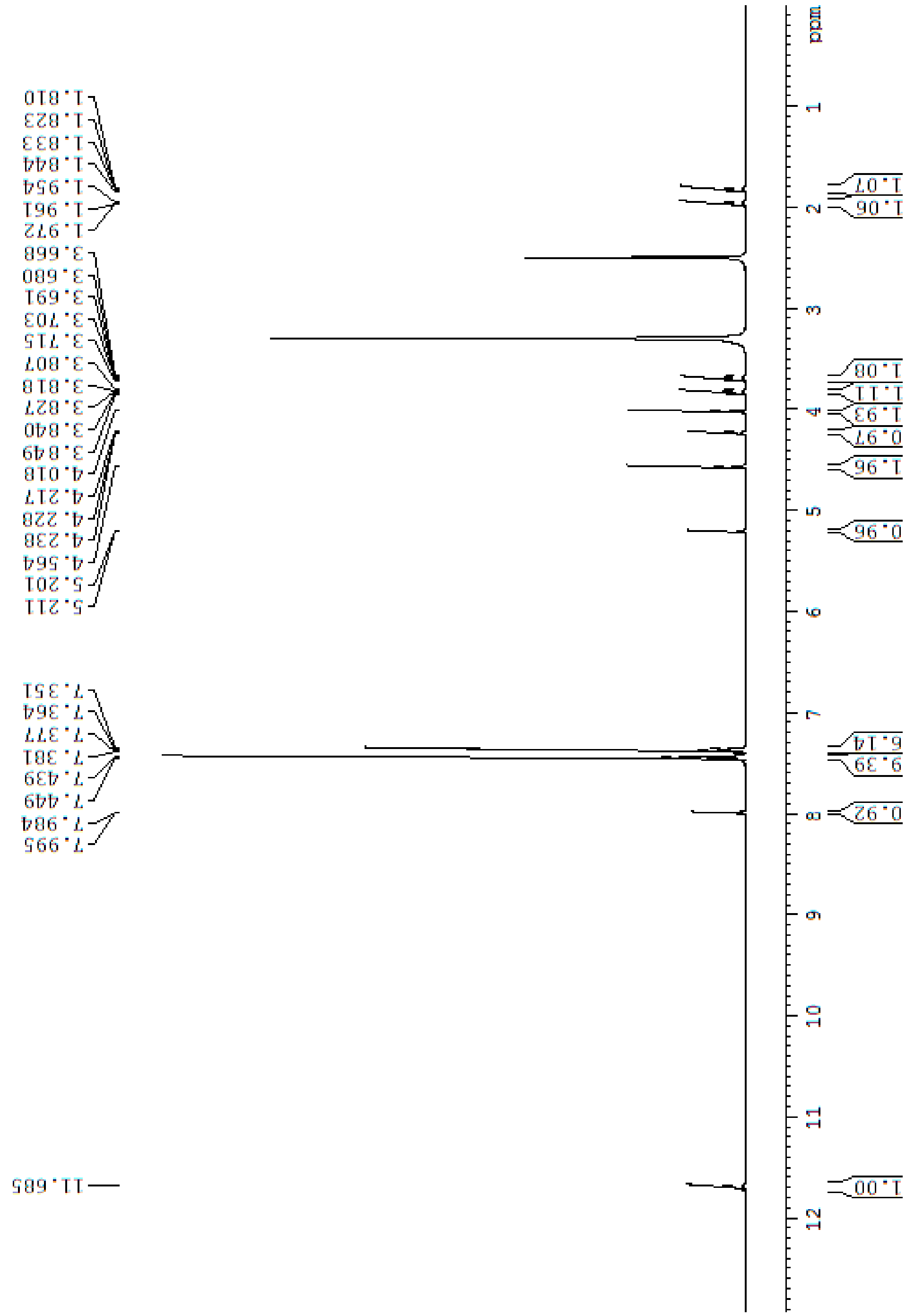

Fig. S3 ^{1}H-NMR of compound **3**.

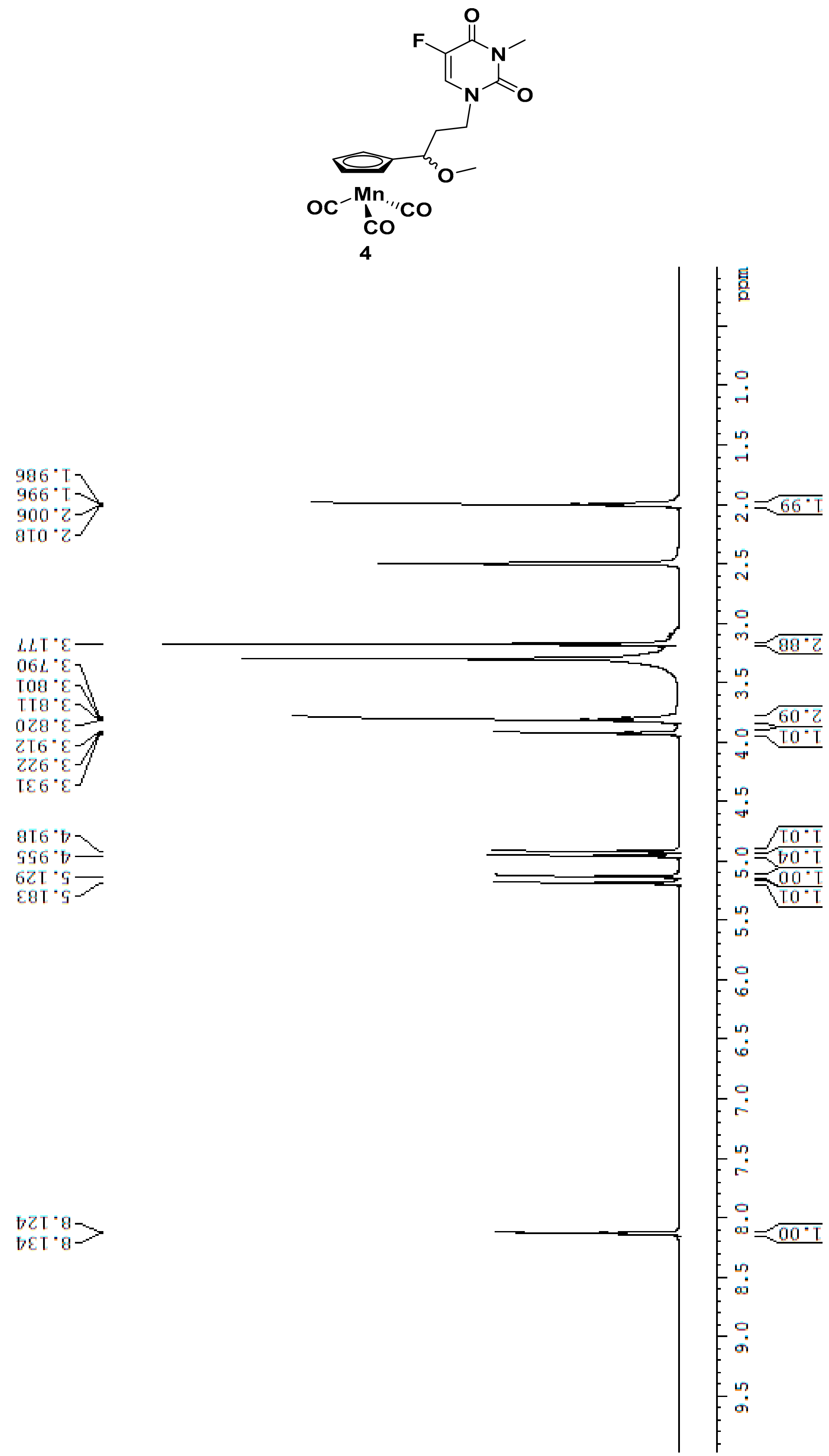

Fig. S4 ^{1}H-NMR of compound **4**.

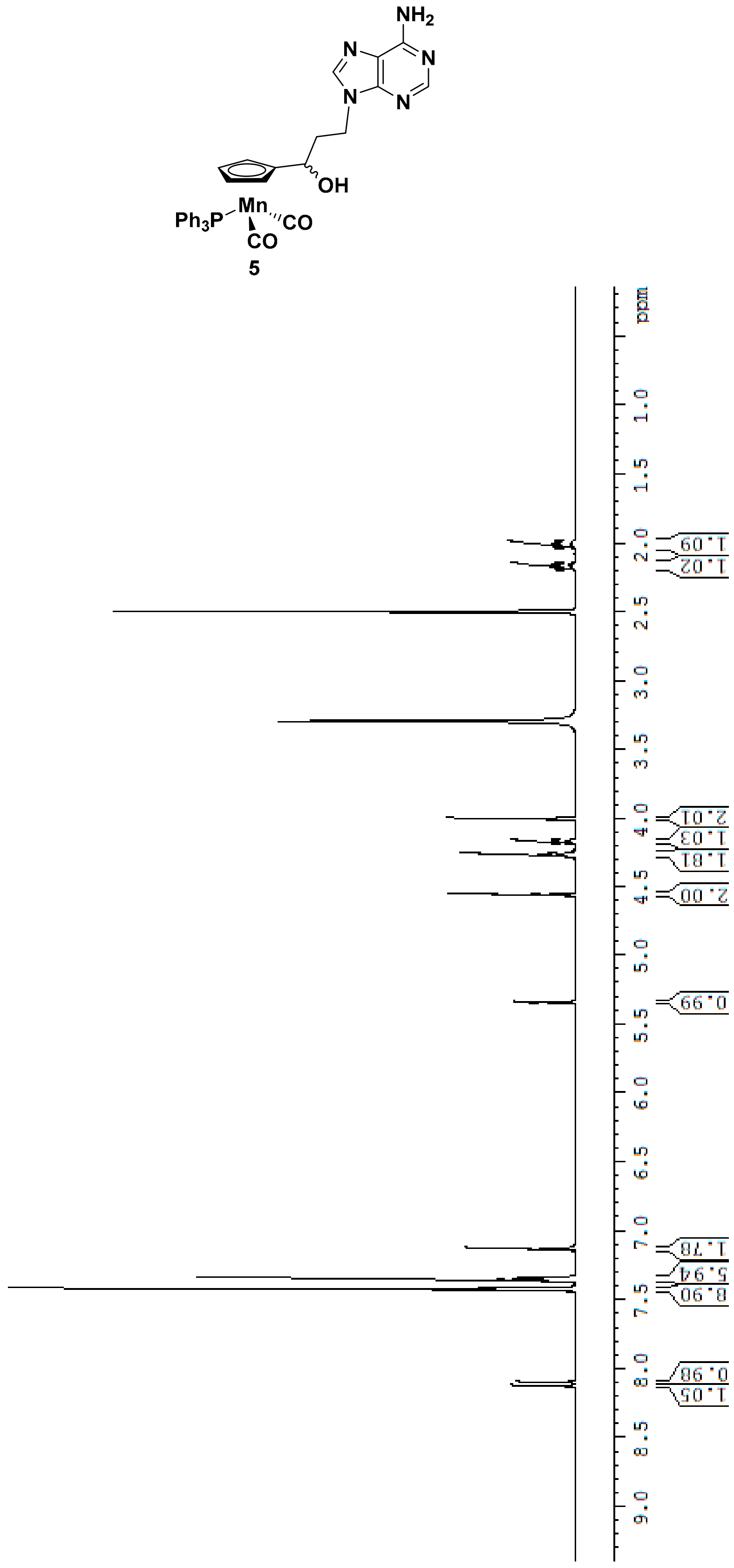

Fig. S5 ^{1}H-NMR of compound **5**.

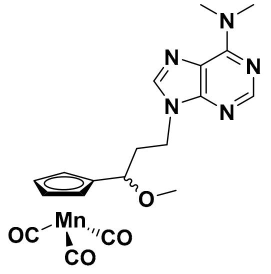

6

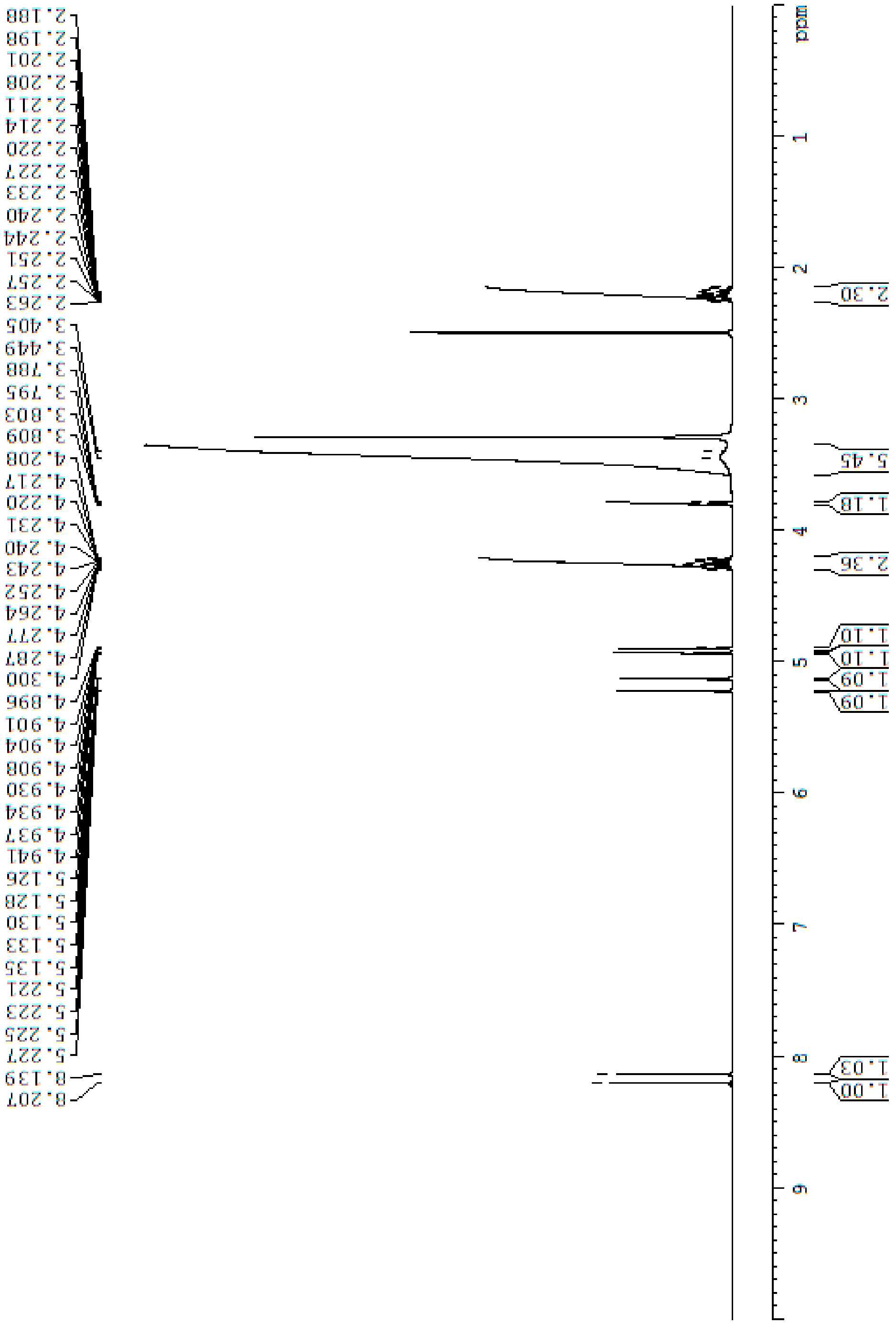

Fig. S6 ^{1}H-NMR of compound **6**.

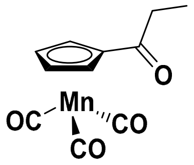

7

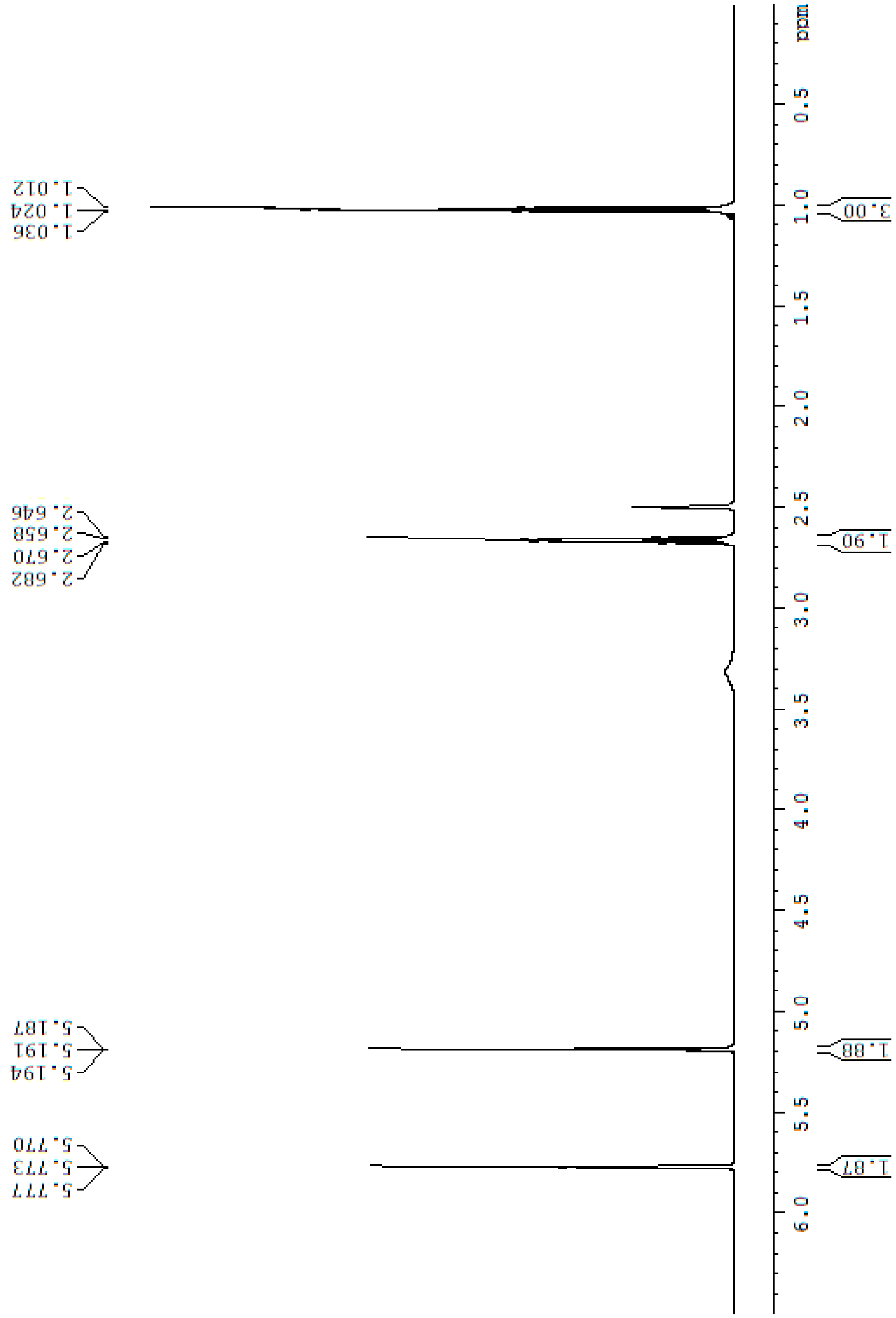

Fig. S7 ^{1}H-NMR of compound **7**.

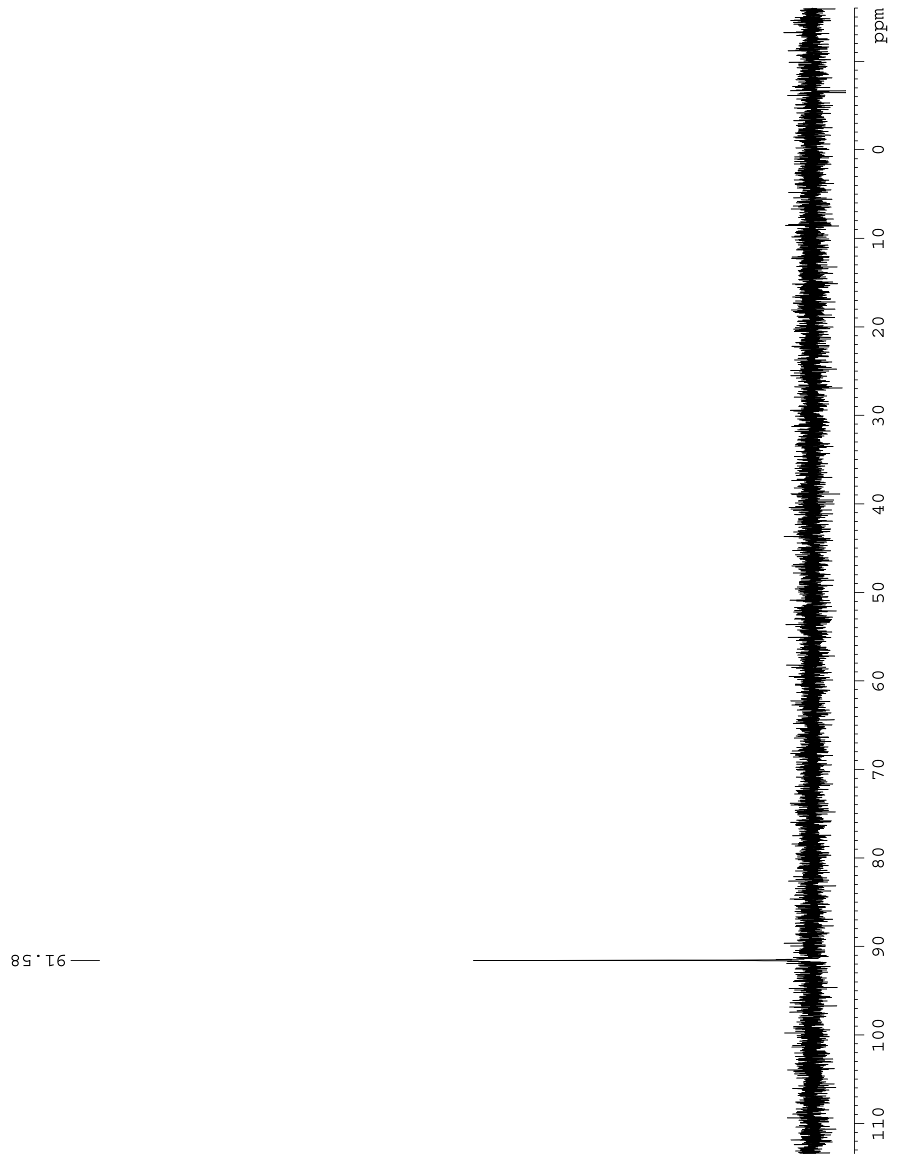

Fig. S8 ^{31}P-NMR of compound **3**

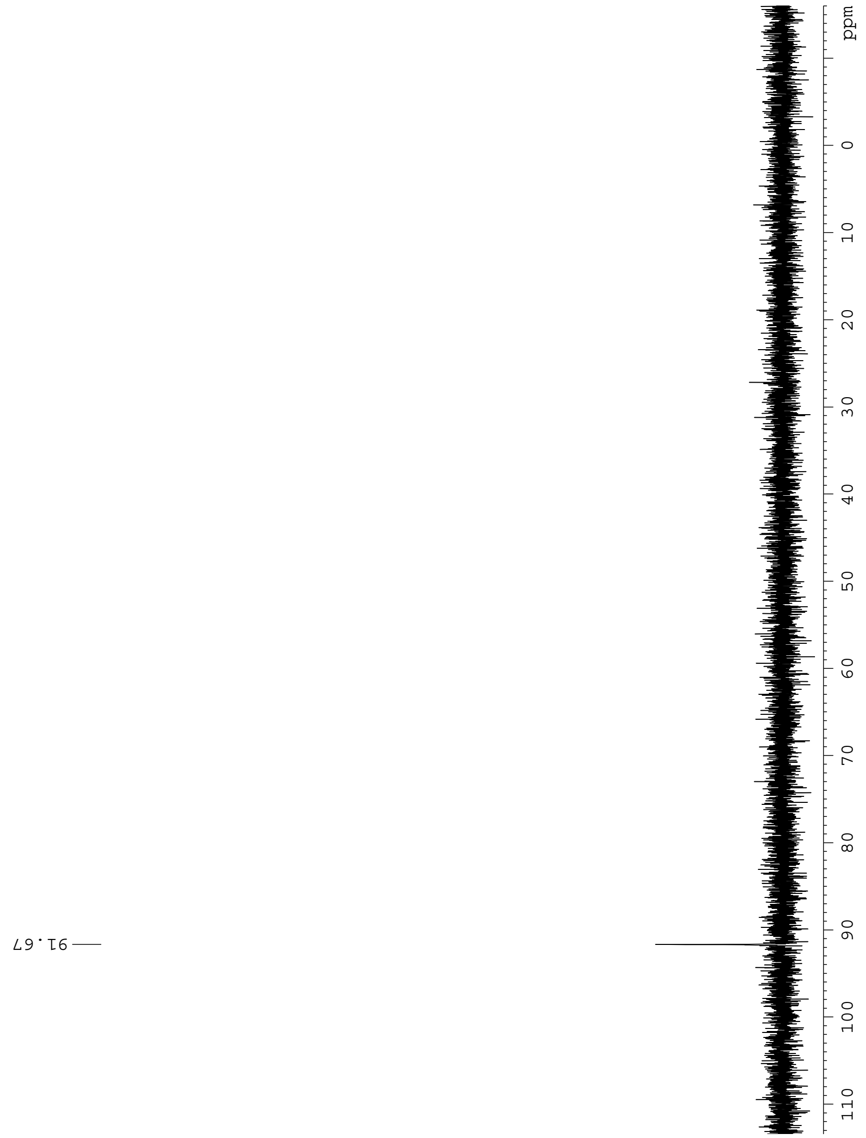

Fig. S9 ^{31}P-NMR of compound **5**

Table S1. Crystallographic data and structural refinement details of 1, 6 and chloroform solvate of C.

**Compound**	**1**	**6**	**C**
Empirical formula	$C_{15}H_{10}FMnN_2O_6$	$C_{19}H_{20}MnN_5O_4$'	$C40H_{38}MnN_5O_2P_2$, $CHCl_3$
Formula weight	388.19	437.34	857.00
Temperature/K	100(2)	100(2)	100(2)
Crystal system	Triclinic	Triclinic	Monoclinic
Space group	P-1	P-1	$P2_1/n$
a/Å	5.8198(3)	7.7371(4)	9.0626(3)
b/Å	12.8370(6)	8.7827(5)	20.9100(6)
c/Å	22.5427(9)	15.1143(10)	21.5509(8)
α/°	100.148(4)	77.475(5)	90
β/°	90.631(4)	89.216(5)	101.667(4)
γ/°	92.995(4)	76.211(5)	90
Volume/Å^3	1655.17(13)	972.94(11)	3999.5(2)
Z	4	2	4
ρ_{calc}/g/cm^3	1.558	1.493	1.423
μ/mm^{-1}	6.917	5.838	5.627
F(000)	784	452	1768
Crystal size/mm	0.41 × 0.06 × 0.03	0.26 × 0.19 × 0.19	0.49 × 0.12 × 0.10
Radiation	Cu$K\alpha$ (λ = 1.54184Å)	Cu$K\alpha$ (λ = 1.54184Å)	Cu$K\alpha$ (λ = 1.54184Å)
2Θ range for data collection/°	3.50 to 74.49	2.99 to 76.70	4.19 to 74.50
Index ranges	$-7 \le h \le 7, -15 \le k \le 16, -21 \le l \le 28$	$-9 \le h \le 9, -9 \le k \le 10, -15 \le l \le 18$	$-11 \le h \le 8, -25 \le k \le 26, -25 \le l \le 26$
Reflections collected	12888	7056	29446
Independent reflections	6757 [R_{int} = 0.0479, R_{sigma} = 0.0766]	3964 [R_{int} = 0.0292, R_{sigma} = 0.0370]	8171 [R_{int} = 0.0339, R_{sigma} = 0.0318]
Data/restraints/parameters	6757/1/ 457	3964/0/266	8171/0/488
Goodness-of-fit on F^2	1.010	1.064	1.044
Final R indexes [I>=2σ (I)]	R_1 = 0.0511, wR_2 = 0.1320	R_1 = 0.0352, wR_2 = 0.0964	R_1 = 0.0589, wR_2 = 0.1661
Final R indexes [all data]	R_1 = 0.0644, wR_2 = 0.1412	R_1 = 0.0364, wR_2 = 0.0978	R_1 = 0.0621, wR_2 = 0.1689
Largest diff. peak/hole / e Å^{-3}	1.10/-0.69	0.83/-0.42	1.01/-1.03

Table S2. Bond lengths for **1A**.

Atom	Atom	Length/Å
C(1A)	Mn(1A)	1.822(4)
C(1A)	O(1A)	1.126(5)
C(2A)	Mn(1A)	1.803(4)
C(2A)	O(2A)	1.147(5)
C(3A)	Mn(1A)	1.796(3)
C(3A)	O(3A)	1.150(4)
C(4A)	C(5A)	1.432(4)
C(4A)	C(8A)	1.442(4)
C(4A)	C(9A)	1.472(4)
C(4A)	Mn(1A)	2.131(3)
C(5A)	C(6A)	1.413(5)
C(5A)	Mn(1A)	2.141(3)
C(6A)	C(7A)	1.421(4)
C(6A)	Mn(1A)	2.158(3)
C(7A)	C(8A)	1.409(4)
C(7A)	Mn(1A)	2.151(3)
C(8A)	Mn(1A)	2.142(3)
C(9A)	C(10A)	1.528(4)
C(9A)	O(4A)	1.220(4)
C(10A)	C(11A)	1.532(4)
C(11A)	N(1A)	1.470(3)
C(12A)	N(1A)	1.369(3)
C(12A)	N(2A)	1.380(4)
C(12A)	O(5A)	1.229(4)
C(13A)	C(14A)	1.441(4)
C(13A)	N(2A)	1.385(3)
C(13A)	O(6A)	1.222(4)
C(14A)	C(15A)	1.336(4)
C(14A)	F(1A)	1.352(3)
C(15A)	N(1A)	1.384(3)
C(1B)	Mn(1B)	1.812(4)
C(1B)	O(1B)	1.145(5)
C(2B)	Mn(1B)	1.805(4)
C(2B)	O(2B)	1.144(5)
C(3B)	Mn(1B)	1.793(3)
C(3B)	O(3B)	1.150(4)
C(4B)	C(5B)	1.435(4)
C(4B)	C(8B)	1.437(4)
C(4B)	C(9B)	1.476(4)
C(4B)	Mn(1B)	2.123(3)
C(5B)	C(6B)	1.410(4)
C(5B)	Mn(1B)	2.144(3)
C(6B)	C(7B)	1.429(4)
C(6B)	Mn(1B)	2.162(3)
C(7B)	C(8B)	1.420(4)
C(7B)	Mn(1B)	2.159(3)
C(8B)	Mn(1B)	2.144(3)
C(9B)	C(10B)	1.525(4)
C(9B)	O(4B)	1.218(4)

C(10B)	C(11B)	1.520(4)
C(11B)	N(1B)	1.474(4)
C(12B)	N(1B)	1.378(4)
C(12B)	N(2B)	1.376(4)
C(12B)	O(5B)	1.228(4)
C(13B)	C(14B)	1.467(4)
C(13B)	N(2B)	1.386(4)
C(13B)	O(6B)	1.209(4)
C(14B)	C(15B)	1.326(5)
C(14B)	F(1B)	1.346(4)
C(15B)	N(1B)	1.384(4)

Table S3. Valence angles for **1B.**

Atom	Atom	Atom	Angle/°
O(1A)	C(1A)	Mn(1A)	179.4(4)
O(2A)	C(2A)	Mn(1A)	179.2(3)
O(3A)	C(3A)	Mn(1A)	177.9(3)
C(5A)	C(4A)	C(8A)	107.5(3)
C(5A)	C(4A)	C(9A)	124.3(3)
C(5A)	C(4A)	Mn(1A)	70.78(16)
C(8A)	C(4A)	C(9A)	128.2(2)
C(8A)	C(4A)	Mn(1A)	70.69(16)
C(9A)	C(4A)	Mn(1A)	122.1(2)
C(4A)	C(5A)	Mn(1A)	70.04(16)
C(6A)	C(5A)	C(4A)	108.1(3)
C(6A)	C(5A)	Mn(1A)	71.45(17)
C(5A)	C(6A)	C(7A)	108.0(3)
C(5A)	C(6A)	Mn(1A)	70.17(18)
C(7A)	C(6A)	Mn(1A)	70.49(18)
C(6A)	C(7A)	Mn(1A)	70.99(19)
C(8A)	C(7A)	C(6A)	109.0(3)
C(8A)	C(7A)	Mn(1A)	70.50(18)
C(4A)	C(8A)	Mn(1A)	69.86(16)
C(7A)	C(8A)	C(4A)	107.4(2)
C(7A)	C(8A)	Mn(1A)	71.18(17)
C(4A)	C(9A)	C(10A)	117.8(3)
O(4A)	C(9A)	C(4A)	120.8(3)
O(4A)	C(9A)	C(10A)	121.4(3)
C(9A)	C(10A)	C(11A)	111.7(2)
N(1A)	C(11A)	C(10A)	111.5(2)
N(1A)	C(12A)	N(2A)	115.2(2)
O(5A)	C(12A)	N(1A)	122.8(3)
O(5A)	C(12A)	N(2A)	122.0(3)
N(2A)	C(13A)	C(14A)	112.5(2)
O(6A)	C(13A)	C(14A)	124.6(2)
O(6A)	C(13A)	N(2A)	122.9(2)
C(15A)	C(14A)	C(13A)	122.3(2)
C(15A)	C(14A)	F(1A)	121.2(2)
F(1A)	C(14A)	C(13A)	116.4(2)
C(14A)	C(15A)	N(1A)	120.5(2)
C(1A)	Mn(1A)	C(4A)	157.67(15)
C(1A)	Mn(1A)	C(5A)	130.84(14)
C(1A)	Mn(1A)	C(6A)	96.80(14)
C(1A)	Mn(1A)	C(7A)	92.84(15)
C(1A)	Mn(1A)	C(8A)	122.44(15)
C(2A)	Mn(1A)	C(1A)	90.01(16)
C(2A)	Mn(1A)	C(4A)	107.89(14)
C(2A)	Mn(1A)	C(5A)	90.23(14)
C(2A)	Mn(1A)	C(6A)	110.03(14)
C(2A)	Mn(1A)	C(7A)	148.54(13)
C(2A)	Mn(1A)	C(8A)	147.15(14)
C(3A)	Mn(1A)	C(1A)	91.97(15)

C(3A)	Mn(1A)	C(2A)	92.07(15)
C(3A)	Mn(1A)	C(4A)	100.38(12)
C(3A)	Mn(1A)	C(5A)	137.13(13)
C(3A)	Mn(1A)	C(6A)	156.12(13)
C(3A)	Mn(1A)	C(7A)	119.11(13)
C(3A)	Mn(1A)	C(8A)	91.75(13)
C(4A)	Mn(1A)	C(5A)	39.18(10)
C(4A)	Mn(1A)	C(6A)	64.98(12)
C(4A)	Mn(1A)	C(7A)	64.91(12)
C(4A)	Mn(1A)	C(8A)	39.45(11)
C(5A)	Mn(1A)	C(6A)	38.38(12)
C(5A)	Mn(1A)	C(7A)	64.59(12)
C(5A)	Mn(1A)	C(8A)	65.51(11)
C(7A)	Mn(1A)	C(6A)	38.52(11)
C(8A)	Mn(1A)	C(6A)	64.82(11)
C(8A)	Mn(1A)	C(7A)	38.32(11)
C(12A)	N(1A)	C(11A)	119.0(2)
C(12A)	N(1A)	C(15A)	121.9(2)
C(15A)	N(1A)	C(11A)	119.0(2)
C(12A)	N(2A)	C(13A)	127.3(2)
O(1B)	C(1B)	Mn(1B)	179.0(4)
O(2B)	C(2B)	Mn(1B)	177.7(3)
O(3B)	C(3B)	Mn(1B)	176.6(3)
C(5B)	C(4B)	C(8B)	107.8(3)
C(5B)	C(4B)	C(9B)	123.7(3)
C(5B)	C(4B)	Mn(1B)	71.16(17)
C(8B)	C(4B)	C(9B)	128.5(2)
C(8B)	C(4B)	Mn(1B)	71.08(18)
C(9B)	C(4B)	Mn(1B)	121.5(2)
C(4B)	C(5B)	Mn(1B)	69.55(16)
C(6B)	C(5B)	C(4B)	108.1(3)
C(6B)	C(5B)	Mn(1B)	71.59(17)
C(5B)	C(6B)	C(7B)	108.3(3)
C(5B)	C(6B)	Mn(1B)	70.21(15)
C(7B)	C(6B)	Mn(1B)	70.56(16)
C(6B)	C(7B)	Mn(1B)	70.83(17)
C(8B)	C(7B)	C(6B)	108.5(3)
C(8B)	C(7B)	Mn(1B)	70.15(17)
C(4B)	C(8B)	Mn(1B)	69.54(17)
C(7B)	C(8B)	C(4B)	107.4(2)
C(7B)	C(8B)	Mn(1B)	71.32(18)
C(4B)	C(9B)	C(10B)	118.5(3)
O(4B)	C(9B)	C(4B)	120.5(3)
O(4B)	C(9B)	C(10B)	121.0(3)
C(11B)	C(10B)	C(9B)	112.9(3)
N(1B)	C(11B)	C(10B)	112.0(3)
N(2B)	C(12B)	N(1B)	115.9(3)
O(5B)	C(12B)	N(1B)	122.0(3)
O(5B)	C(12B)	N(2B)	122.1(3)
N(2B)	C(13B)	C(14B)	111.0(3)
O(6B)	C(13B)	C(14B)	126.1(3)

O(6B)	C(13B)	N(2B)	122.9(3)
C(15B)	C(14B)	C(13B)	122.7(3)
C(15B)	C(14B)	F(1B)	121.5(3)
F(1B)	C(14B)	C(13B)	115.7(3)
C(14B)	C(15B)	N(1B)	121.4(3)
C(1B)	Mn(1B)	C(4B)	157.49(14)
C(1B)	Mn(1B)	C(5B)	131.45(13)
C(1B)	Mn(1B)	C(6B)	97.27(14)
C(1B)	Mn(1B)	C(7B)	92.44(15)
C(1B)	Mn(1B)	C(8B)	121.85(15)
C(2B)	Mn(1B)	C(1B)	93.13(17)
C(2B)	Mn(1B)	C(4B)	105.38(14)
C(2B)	Mn(1B)	C(5B)	88.02(13)
C(2B)	Mn(1B)	C(6B)	108.73(13)
C(2B)	Mn(1B)	C(7B)	147.34(13)
C(2B)	Mn(1B)	C(8B)	144.60(14)
C(3B)	Mn(1B)	C(1B)	92.46(14)
C(3B)	Mn(1B)	C(2B)	91.32(14)
C(3B)	Mn(1B)	C(4B)	99.68(13)
C(3B)	Mn(1B)	C(5B)	136.07(14)
C(3B)	Mn(1B)	C(6B)	157.08(14)
C(3B)	Mn(1B)	C(7B)	120.55(13)
C(3B)	Mn(1B)	C(8B)	92.33(13)
C(4B)	Mn(1B)	C(5B)	39.29(10)
C(4B)	Mn(1B)	C(6B)	64.98(12)
C(4B)	Mn(1B)	C(7B)	65.06(12)
C(4B)	Mn(1B)	C(8B)	39.37(11)
C(5B)	Mn(1B)	C(6B)	38.21(12)
C(5B)	Mn(1B)	C(7B)	64.63(12)
C(7B)	Mn(1B)	C(6B)	38.61(11)
C(8B)	Mn(1B)	C(5B)	65.52(11)
C(8B)	Mn(1B)	C(6B)	64.91(12)
C(8B)	Mn(1B)	C(7B)	38.53(11)
C(12B)	N(1B)	C(11B)	119.3(3)
C(12B)	N(1B)	C(15B)	120.7(3)
C(15B)	N(1B)	C(11B)	120.1(3)
C(12B)	N(2B)	C(13B)	128.3(2)

Table S4. Bond lengths for **6**.

Atom	Atom	Length/Å
C(3)	Mn(1)	1.797(2)
C(3)	O(3)	1.150(3)
C(2)	Mn(1)	1.789(2)
C(2)	O(2)	1.152(3)
C(1)	Mn(1)	1.7950(19)
C(1)	O(1)	1.151(2)
C(4)	C(5)	1.429(2)
C(4)	C(8)	1.423(2)
C(4)	C(9)	1.503(2)
C(4)	Mn(1)	2.1449(17)
C(5)	C(6)	1.411(3)
C(5)	Mn(1)	2.1457(19)
C(6)	C(7)	1.416(3)
C(6)	Mn(1)	2.144(2)
C(7)	C(8)	1.416(3)
C(7)	Mn(1)	2.142(2)
C(8)	Mn(1)	2.1376(18)
C(9)	C(10)	1.536(2)
C(9)	O(4)	1.412(2)
C(10)	C(11)	1.522(2)
C(11)	N(1)	1.463(2)
C(12)	N(1)	1.368(2)
C(12)	N(2)	1.311(2)
C(13)	C(14)	1.418(2)
C(13)	C(16)	1.392(2)
C(13)	N(2)	1.396(2)
C(14)	N(3)	1.358(2)
C(14)	N(5)	1.353(2)
C(15)	N(3)	1.342(2)
C(15)	N(4)	1.329(2)
C(16)	N(1)	1.369(2)
C(16)	N(4)	1.346(2)
C(17)	N(5)	1.460(2)
C(18)	N(5)	1.460(2)
C(19)	O(4)	1.423(2)

Table S5. Valence angles for **6**.

Atom	Atom	Atom	Angle/°
O(3)	C(3)	Mn(1)	178.83(18)
O(2)	C(2)	Mn(1)	179.49(19)
O(1)	C(1)	Mn(1)	179.15(19)
C(5)	C(4)	C(9)	124.80(16)
C(5)	C(4)	Mn(1)	70.58(10)
C(8)	C(4)	C(5)	106.93(16)
C(8)	C(4)	C(9)	127.97(16)
C(8)	C(4)	Mn(1)	70.32(10)
C(9)	C(4)	Mn(1)	129.07(12)
C(4)	C(5)	Mn(1)	70.52(10)
C(6)	C(5)	C(4)	108.51(17)
C(6)	C(5)	Mn(1)	70.75(11)
C(5)	C(6)	C(7)	108.05(17)
C(5)	C(6)	Mn(1)	70.85(11)
C(7)	C(6)	Mn(1)	70.62(11)
C(6)	C(7)	Mn(1)	70.81(12)
C(8)	C(7)	C(6)	108.04(18)
C(8)	C(7)	Mn(1)	70.52(11)
C(4)	C(8)	Mn(1)	70.87(10)
C(7)	C(8)	C(4)	108.46(17)
C(7)	C(8)	Mn(1)	70.84(11)
C(4)	C(9)	C(10)	111.31(15)
O(4)	C(9)	C(4)	111.32(15)
O(4)	C(9)	C(10)	107.79(14)
C(11)	C(10)	C(9)	111.63(14)
N(1)	C(11)	C(10)	112.69(14)
N(2)	C(12)	N(1)	113.82(15)
C(16)	C(13)	C(14)	115.87(15)
C(16)	C(13)	N(2)	109.78(14)
N(2)	C(13)	C(14)	134.33(16)
N(3)	C(14)	C(13)	117.09(16)
N(5)	C(14)	C(13)	125.09(16)
N(5)	C(14)	N(3)	117.77(15)
N(4)	C(15)	N(3)	129.33(17)
N(1)	C(16)	C(13)	106.17(15)
N(4)	C(16)	C(13)	128.48(16)
N(4)	C(16)	N(1)	125.34(16)
C(3)	Mn(1)	C(4)	101.54(8)
C(3)	Mn(1)	C(5)	139.34(8)
C(3)	Mn(1)	C(6)	152.46(9)
C(3)	Mn(1)	C(7)	114.53(9)
C(3)	Mn(1)	C(8)	89.56(8)
C(2)	Mn(1)	C(3)	90.87(9)
C(2)	Mn(1)	C(1)	92.11(9)
C(2)	Mn(1)	C(4)	104.27(8)
C(2)	Mn(1)	C(5)	91.71(8)
C(2)	Mn(1)	C(6)	115.23(9)

C(2)	Mn(1)	C(7)	153.55(9)
C(2)	Mn(1)	C(8)	141.92(8)
C(1)	Mn(1)	C(3)	92.64(9)
C(1)	Mn(1)	C(4)	158.00(8)
C(1)	Mn(1)	C(5)	127.78(8)
C(1)	Mn(1)	C(6)	94.90(8)
C(1)	Mn(1)	C(7)	93.89(8)
C(1)	Mn(1)	C(8)	125.90(8)
C(4)	Mn(1)	C(5)	38.90(7)
C(6)	Mn(1)	C(4)	65.00(7)
C(6)	Mn(1)	C(5)	38.39(8)
C(7)	Mn(1)	C(4)	65.00(7)
C(7)	Mn(1)	C(5)	64.48(8)
C(7)	Mn(1)	C(6)	38.57(9)
C(8)	Mn(1)	C(4)	38.81(6)
C(8)	Mn(1)	C(5)	64.68(7)
C(8)	Mn(1)	C(6)	64.70(8)
C(8)	Mn(1)	C(7)	38.63(7)
C(12)	N(1)	C(11)	128.66(15)
C(12)	N(1)	C(16)	106.11(14)
C(16)	N(1)	C(11)	125.18(14)
C(12)	N(2)	C(13)	104.11(14)
C(15)	N(3)	C(14)	119.46(16)
C(15)	N(4)	C(16)	109.73(15)
C(14)	N(5)	C(17)	121.40(15)
C(14)	N(5)	C(18)	119.80(15)
C(17)	N(5)	C(18)	115.15(16)
C(9)	O(4)	C(19)	113.69(15)

Table S6. Bond lengths for chloroform solvate of **C**.

Atom	Atom	Length/Å
C(1)	Mn(1)	1.760(3)
C(1)	O(1)	1.169(4)
C(2)	C(3)	1.540(4)
C(2)	P(1)	1.854(3)
C(3)	P(2)	1.862(3)
C(4)	C(5)	1.397(5)
C(4)	C(9)	1.391(5)
C(4)	P(1)	1.834(3)
C(5)	C(6)	1.382(5)
C(6)	C(7)	1.376(6)
C(7)	C(8)	1.388(7)
C(8)	C(9)	1.395(5)
C(10)	C(11)	1.385(5)
C(10)	C(15)	1.406(5)
C(10)	P(1)	1.844(3)
C(11)	C(12)	1.398(5)
C(12)	C(13)	1.364(7)
C(13)	C(14)	1.386(7)
C(14)	C(15)	1.399(6)
C(16)	C(17)	1.404(5)
C(16)	C(21)	1.397(5)
C(16)	P(2)	1.836(3)
C(17)	C(18)	1.390(5)
C(18)	C(19)	1.382(6)
C(19)	C(20)	1.390(6)
C(20)	C(21)	1.389(5)
C(22)	C(23)	1.387(5)
C(22)	C(27)	1.408(5)
C(22)	P(2)	1.844(4)
C(23)	C(24)	1.399(6)
C(24)	C(25)	1.390(7)
C(25)	C(26)	1.398(7)
C(26)	C(27)	1.380(6)
C(28)	C(29)	1.421(5)
C(28)	C(32)	1.423(4)
C(28)	C(33)	1.502(4)
C(28)	Mn(1)	2.135(3)
C(29)	C(30)	1.425(5)
C(29)	Mn(1)	2.124(3)
C(30)	C(31)	1.424(5)
C(30)	Mn(1)	2.133(3)
C(31)	C(32)	1.412(5)
C(31)	Mn(1)	2.152(3)
C(32)	Mn(1)	2.159(3)
C(33)	C(34)	1.521(4)
C(33)	O(2)	1.417(4)

C(34)	C(35)	1.516(4)
C(35)	N(1)	1.465(4)
C(36)	N(1)	1.356(4)
C(36)	N(2)	1.313(4)
C(37)	C(38)	1.413(4)
C(37)	C(40)	1.381(4)
C(37)	N(2)	1.379(4)
C(38)	N(3)	1.354(4)
C(38)	N(5)	1.327(4)
C(39)	N(3)	1.337(4)
C(39)	N(4)	1.332(4)
C(40)	N(1)	1.371(4)
C(40)	N(4)	1.343(4)
Mn(1)	P(1)	2.1990(9)
Mn(1)	P(2)	2.1933(9)
C(41)	Cl(1)	1.730(7)
C(41)	Cl(2)	1.773(7)
C(41)	Cl(3)	1.762(5)

Table S7. Valence angles for chloroform solvate of **C**.

Atom	Atom	Atom	Angle/°
O(1)	C(1)	Mn(1)	177.4(3)
C(3)	C(2)	P(1)	107.5(2)
C(2)	C(3)	P(2)	109.8(2)
C(5)	C(4)	P(1)	118.6(3)
C(9)	C(4)	C(5)	118.0(3)
C(9)	C(4)	P(1)	123.3(3)
C(6)	C(5)	C(4)	121.7(4)
C(7)	C(6)	C(5)	120.0(4)
C(6)	C(7)	C(8)	119.4(4)
C(7)	C(8)	C(9)	120.7(4)
C(4)	C(9)	C(8)	120.2(4)
C(11)	C(10)	C(15)	118.8(3)
C(11)	C(10)	P(1)	119.3(3)
C(15)	C(10)	P(1)	121.9(3)
C(10)	C(11)	C(12)	120.8(4)
C(13)	C(12)	C(11)	120.2(4)
C(12)	C(13)	C(14)	120.2(4)
C(13)	C(14)	C(15)	120.3(4)
C(14)	C(15)	C(10)	119.7(4)
C(17)	C(16)	P(2)	118.2(3)
C(21)	C(16)	C(17)	118.0(3)
C(21)	C(16)	P(2)	122.8(3)
C(18)	C(17)	C(16)	120.6(3)
C(19)	C(18)	C(17)	120.6(4)
C(18)	C(19)	C(20)	119.5(3)
C(21)	C(20)	C(19)	120.2(3)
C(20)	C(21)	C(16)	121.1(3)
C(23)	C(22)	C(27)	118.2(4)
C(23)	C(22)	P(2)	121.1(3)
C(27)	C(22)	P(2)	120.6(3)
C(22)	C(23)	C(24)	121.1(4)
C(25)	C(24)	C(23)	119.9(4)
C(24)	C(25)	C(26)	119.6(4)
C(27)	C(26)	C(25)	120.0(4)
C(26)	C(27)	C(22)	121.1(4)
C(29)	C(28)	C(32)	107.3(3)
C(29)	C(28)	C(33)	129.9(3)
C(29)	C(28)	Mn(1)	70.08(18)
C(32)	C(28)	C(33)	122.4(3)
C(32)	C(28)	Mn(1)	71.54(17)
C(33)	C(28)	Mn(1)	129.4(2)
C(28)	C(29)	C(30)	108.7(3)
C(28)	C(29)	Mn(1)	70.95(18)
C(30)	C(29)	Mn(1)	70.80(18)
C(29)	C(30)	Mn(1)	70.07(18)
C(31)	C(30)	C(29)	107.1(3)
C(31)	C(30)	Mn(1)	71.30(19)
C(30)	C(31)	Mn(1)	69.89(18)

C(32)	C(31)	C(30)	108.5(3)
C(32)	C(31)	Mn(1)	71.13(17)
C(28)	C(32)	Mn(1)	69.75(18)
C(31)	C(32)	C(28)	108.4(3)
C(31)	C(32)	Mn(1)	70.61(18)
C(28)	C(33)	C(34)	114.8(3)
O(2)	C(33)	C(28)	109.7(3)
O(2)	C(33)	C(34)	107.9(2)
C(35)	C(34)	C(33)	108.5(3)
N(1)	C(35)	C(34)	113.6(3)
N(2)	C(36)	N(1)	114.0(3)
C(40)	C(37)	C(38)	116.9(3)
N(2)	C(37)	C(38)	132.4(3)
N(2)	C(37)	C(40)	110.7(2)
N(3)	C(38)	C(37)	116.8(3)
N(5)	C(38)	C(37)	124.4(3)
N(5)	C(38)	N(3)	118.7(3)
N(4)	C(39)	N(3)	128.9(3)
N(1)	C(40)	C(37)	105.7(2)
N(4)	C(40)	C(37)	127.4(3)
N(4)	C(40)	N(1)	126.9(3)
C(1)	Mn(1)	C(28)	102.26(13)
C(1)	Mn(1)	C(29)	91.38(14)
C(1)	Mn(1)	C(30)	116.87(15)
C(1)	Mn(1)	C(31)	154.83(15)
C(1)	Mn(1)	C(32)	139.41(13)
C(1)	Mn(1)	P(1)	85.94(11)
C(1)	Mn(1)	P(2)	92.32(11)
C(28)	Mn(1)	C(31)	64.90(12)
C(28)	Mn(1)	C(32)	38.71(11)
C(28)	Mn(1)	P(1)	108.28(10)
C(28)	Mn(1)	P(2)	160.43(9)
C(29)	Mn(1)	C(28)	38.97(14)
C(29)	Mn(1)	C(30)	39.12(12)
C(29)	Mn(1)	C(31)	64.82(12)
C(29)	Mn(1)	C(32)	64.66(13)
C(29)	Mn(1)	P(1)	145.53(10)
C(29)	Mn(1)	P(2)	128.99(11)
C(30)	Mn(1)	C(28)	65.63(14)
C(30)	Mn(1)	C(31)	38.81(14)
C(30)	Mn(1)	C(32)	64.84(15)
C(30)	Mn(1)	P(1)	156.95(11)
C(30)	Mn(1)	P(2)	96.22(10)
C(31)	Mn(1)	C(32)	38.25(13)
C(31)	Mn(1)	P(1)	118.15(11)
C(31)	Mn(1)	P(2)	96.56(9)
C(32)	Mn(1)	P(1)	96.10(10)
C(32)	Mn(1)	P(2)	128.27(9)
P(2)	Mn(1)	P(1)	85.48(3)
C(36)	N(1)	C(35)	127.8(3)
C(36)	N(1)	C(40)	106.0(2)

C(40)	N(1)	C(35)	125.5(2)
C(36)	N(2)	C(37)	103.7(2)
C(39)	N(3)	C(38)	119.5(3)
C(39)	N(4)	C(40)	110.4(3)
C(2)	P(1)	Mn(1)	107.34(11)
C(4)	P(1)	C(2)	104.66(15)
C(4)	P(1)	C(10)	101.02(16)
C(4)	P(1)	Mn(1)	117.02(11)
C(10)	P(1)	C(2)	102.27(15)
C(10)	P(1)	Mn(1)	122.40(12)
C(3)	P(2)	Mn(1)	111.04(11)
C(16)	P(2)	C(3)	105.21(15)
C(16)	P(2)	C(22)	100.22(15)
C(16)	P(2)	Mn(1)	114.60(10)
C(22)	P(2)	C(3)	103.26(16)
C(22)	P(2)	Mn(1)	120.76(12)
Cl(1)	C(41)	Cl(2)	111.1(3)
Cl(1)	C(41)	Cl(3)	112.4(4)
Cl(3)	C(41)	Cl(2)	107.8(3)

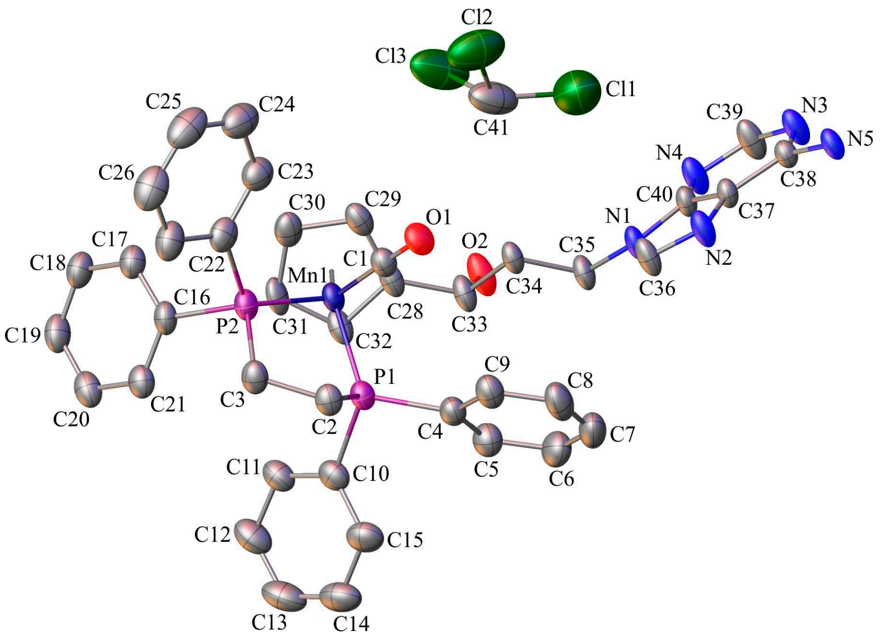

Fig. S10 The asymmetric part of the unit cell of the chloroform solvate of **C** with crystallographic numbering. Displacement ellipsoids are drawn at the 50% probability level. The hydrogen atoms were omitted for clarity.

Table S8. Equations, R^2 coefficients and log $P_{o/w}$ values for the studied compounds.

**Compound**	**Equation**	**R^2**	**log $P_{o/w}$**
**1**	**y = -0.0432x + 2.5857**	**0.9950**	**2.6**
**2**	**y = -0.0444x + 2.8034**	**0.9949**	**2.8**
**3**	**y = -0.063x + 5.246**	**0.9996**	**5.2**
**4**	**y = -0,0466x + 3,4939**	**0.9976**	**3.5**
**5**	**y = -0.0569x + 4.8522**	**0.9982**	**4.8**
**6**	**y = -0,0418x + 3,4585**	**0.9966**	**3.5**
7	**y = -0.0428x + 3.2356**	**0.9925**	**3.2**
**CymH**	**y = -0.0443x + 3.5131**	**0.9967**	**3.5**
**B**	**y = -0.0379x + 2.5506**	**0.9972**	**2.6**
**C**	**y = -0.0612x + 5.677**	**0.9981**	**5.7**

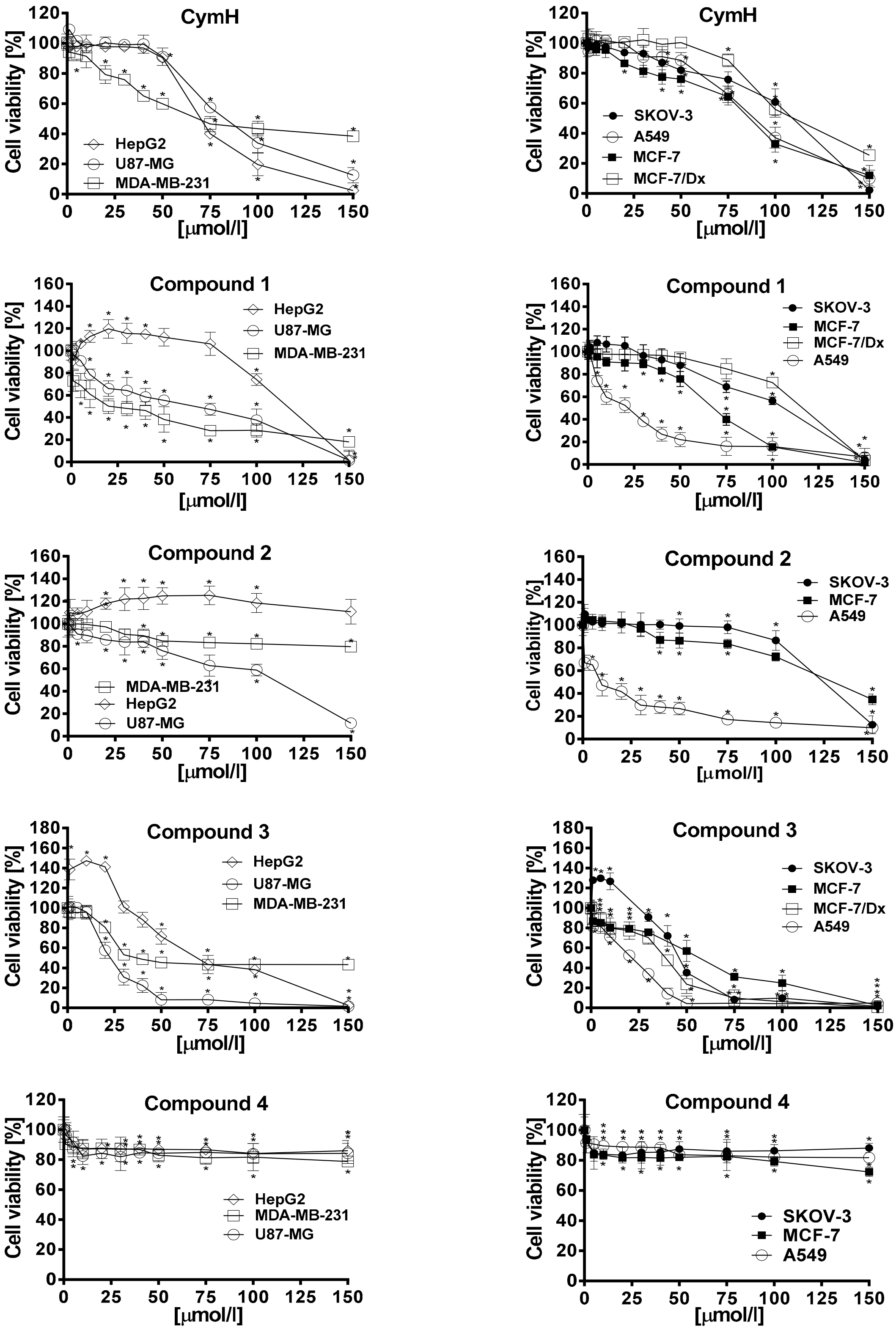

Fig. S11A Survival curves of human ovarian (SKOV-3), breast (MCF-7, MCF-7/DX, MDA-MB-231), lung (A549), liver (HepG2) and brain (U87-MG) cancer cells treated for 24 h with cymantrenes **CymH** and **1-4**. Results are expressed as means ± SD of at least three independent experiments in six repeats each. *$P<0.05$ relative to untreated cells (control).

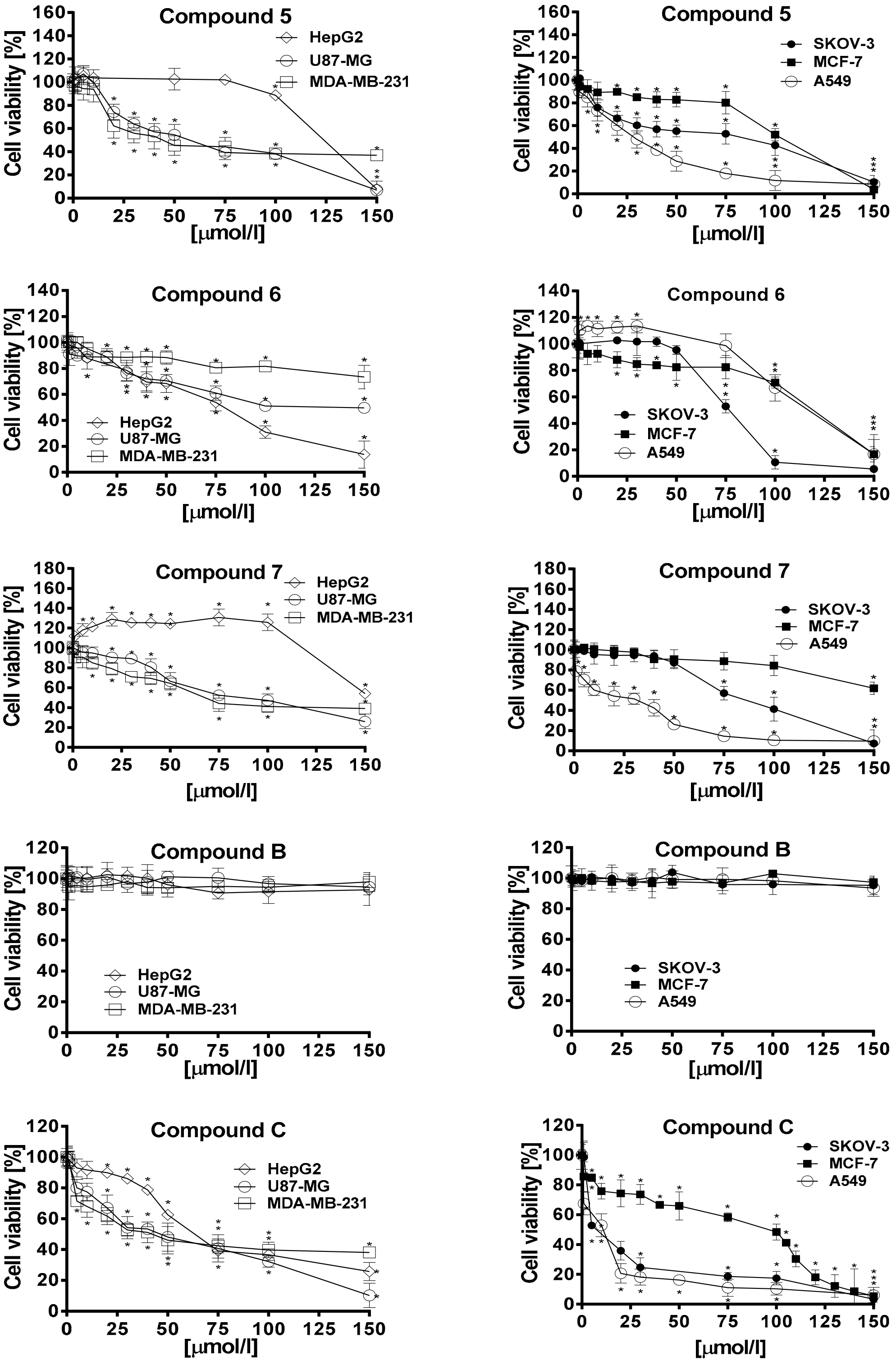

Fig. S11B Survival curves of human ovarian (SKOV-3), breast (MCF-7, MCF-7/DX, MDA-MB-231), lung (A549), liver (HepG2) and brain (U87-MG) cancer cells treated for 24 h with cymantrenes **5-7, B** and **C.** Results are expressed as means ± SD of at least three independent experiments in six repeats each. *P<0.05 relative to untreated cells (control).

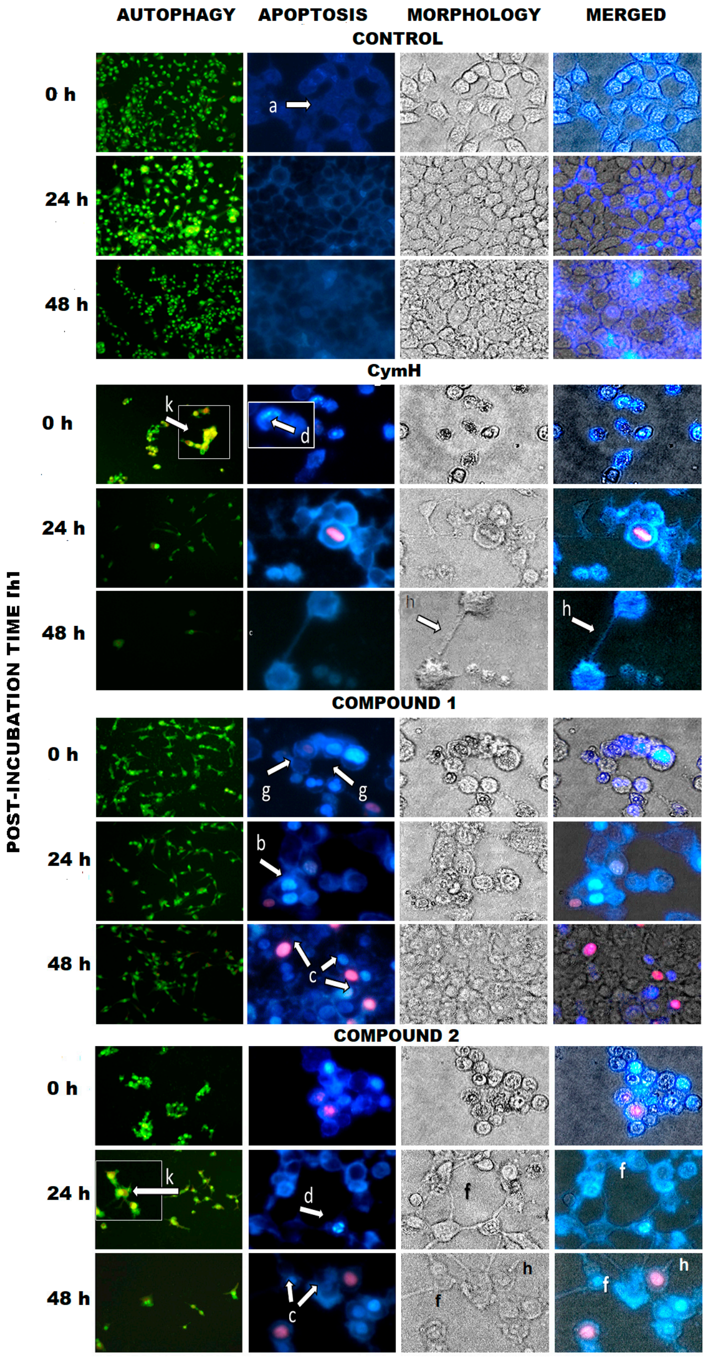

Fig. S12A Example photomicrograph illustrating induction of apoptosis and autophagy in human ovarian adenocarcinoma cells SKOV-3 treated with cymantrenes **CymH**, **1** and **2** for 24 h and then cultured in drug-free medium for 24 or 48 h. 0 h – cells examined immediately after the treatment. The images from left to right show cells stained with acridine orange (identification of acidic vesicular organelles (AVOs), a hallmark of autophagy), cells double-stained with fluorescence dyes Hoechst 33258 (Ho33258) and propidium iodide (PI), unstained cells and merged images of unstained and Ho33258/PI stained cells. Cells were analyzed with an inverted fluorescence microscope (Olympus IX70, Japan) at 400×magnification, except cells stained with acridine orange (autophagy panel), which were photographed under 150×magnification. Morphological changes marked with the arrows: **(a)** pale-blue live cells; (**b**) intense bright-blue early apoptotic shrunk cells with pycnotic nucleus with highly condensed and **(d)** fragmented chromatin (karyorrhexis); **(c)** violet late apoptotic cells; **(e)** marginalization of chromatin; **(f)** cells with plasma membrane protrusions ("blebs") and **(g)** apoptotic bodies; **(h)** cytoplasmic bridges between the cells. Giant cells typical for mitotic catastrophe (**i**); polyploid cells with two nuclei **(j)** and cells with AVOs **(k)** suggest concomitant mitotic catastrophe and autophagy. Red necrotic cells are not present in the example images.

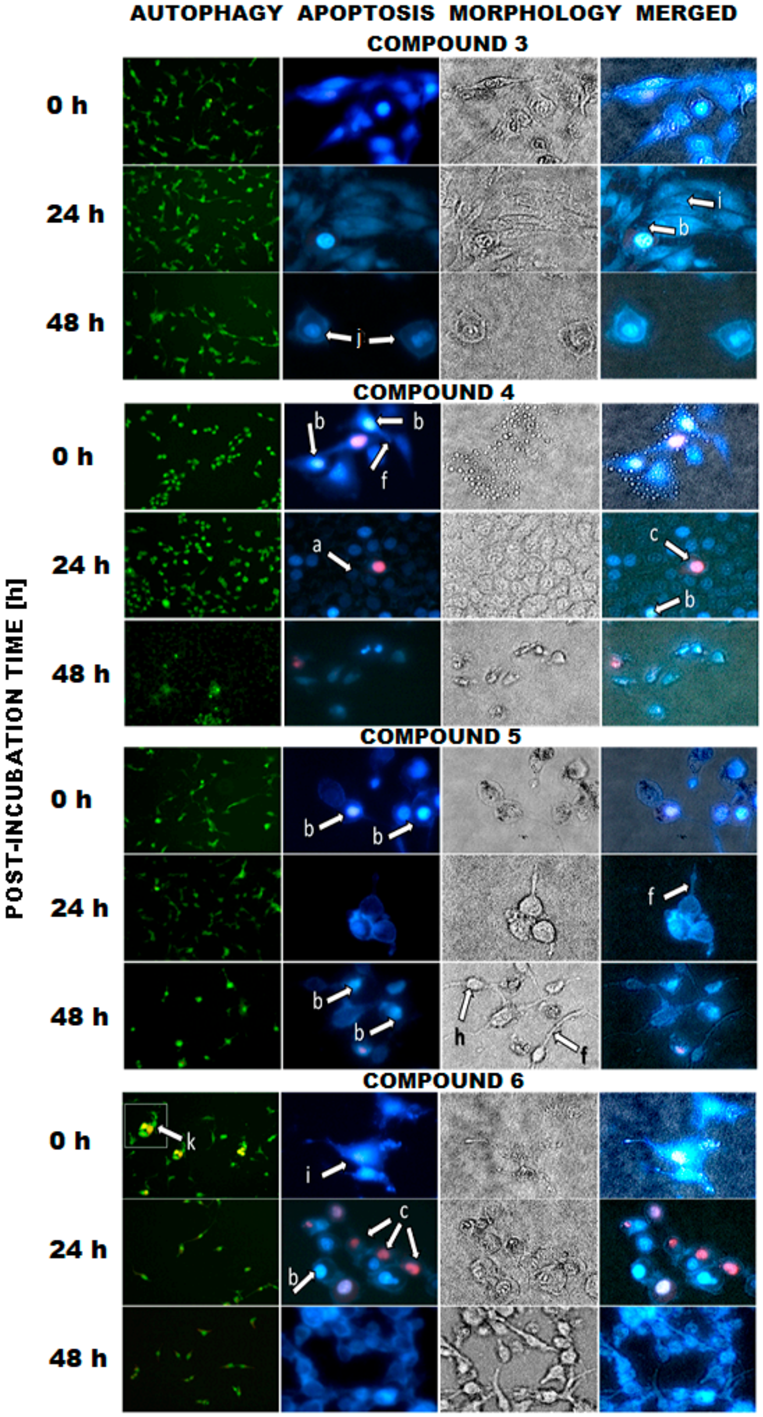

Fig. S12B. Example photomicrograph illustrating induction of apoptosis and autophagy in human ovarian adenocarcinoma cells SKOV-3 treated with cymantrenes **3, 4, 5** and **6** for 24 h and then cultured in drug-free medium for 24 or 48 h. 0 h – cells examined immediately after the treatment. The images from left to right show cells stained with acridine orange (identification of acidic vesicular organelles (AVOs), a hallmark of autophagy), cells double-stained with fluorescence dyes Hoechst 33258 (Ho33258) and propidium iodide (PI), unstained cells and merged images of unstained and Ho33258/PI stained cells. Cells were analyzed with an inverted fluorescence microscope (Olympus IX70, Japan) at 400×magnification, except cells stained with acridine orange (autophagy panel), which were photographed under 150×magnification. Morphological changes marked with the arrows: **(a)** pale-blue live cells; (**b**) intense bright-blue shrunk early apoptotic cells with pycnotic nucleus and highly condensed chromatin; **(c)** violet late apoptotic cells; **(f)** cells with plasma membrane protrusions (“blebs”); **(h)** cytoplasmic bridges between the cells; **(i)** giant and polyploidy **(j)** cells typical for mitotic catastrophe **(k)** orange autophagic cells with AVOs.

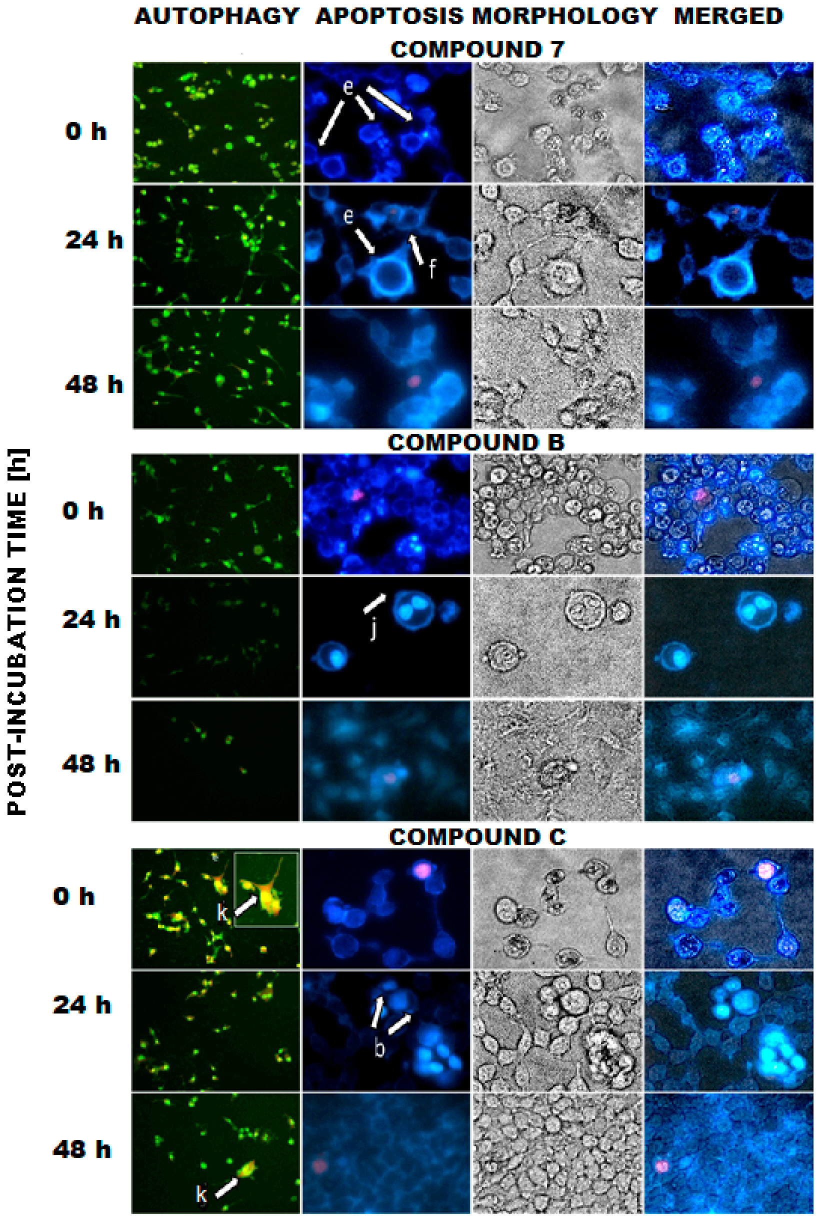

Fig. S12C Example photomicrograph illustrating induction of apoptosis and autophagy in human ovarian adenocarcinoma cells SKOV-3 treated with cymantrenes **7, B** and **C** for 24 h and then cultured in drug-free medium for 24 or 48 h. 0 h – cells examined immediately after the treatment. The images from left to right show cells stained with acridine orange (identification of acidic vesicular organelles (AVOs), a hallmark of autophagy), cells double-stained with fluorescence dyes Hoechst 33258 (Ho33258) and propidium iodide (PI), unstained cells and merged images of unstained and Ho33258/PI stained cells. Cells were analyzed with an inverted fluorescence microscope (Olympus IX70, Japan) at 400×magnification, except cells stained with acridine orange (autophagy panel), which were photographed under 150×magnification. Morphological changes marked with the arrows: (**b**) shrunk cells with pycnotic nucleus and highly condensed chromatin; **(e)** marginalization of chromatin; **(i)** giant cells and **(j)** polyploid cells with two nuclei typical for mitotic catastrophe; (k) orange autophagic cells with AVOs.
